# Immunological reference intervals in pregnancy: longitudinal analysis of adaptive lymphocyte subsets

**DOI:** 10.3389/fimmu.2025.1634176

**Published:** 2025-09-17

**Authors:** Miguel Ângelo-Dias, Catarina Gregório Martins, Mariana Mata, Madalena Barata, Ana Chung, Susana Sarzedas, Élia Fernandes, Cláudia Appleton, Jorge Lima, Luis Miguel Borrego

**Affiliations:** ^1^ Immunology Department, NOVA Medical School, Faculdade de Ciências Médicas, NMS, FCM, Universidade NOVA de Lisboa, Lisbon, Portugal; ^2^ Comprehensive Health Research Centre – CHRC, NOVA Medical School, Faculdade de Ciências Médicas, NMS, FCM, Universidade NOVA de Lisboa, Lisbon, Portugal; ^3^ Department of Obstetrics and Gynecology, High-Risk Pregnancy Center, Hospital da Luz Lisboa, Lisbon, Portugal; ^4^ Department of Immunoallergy, Hospital da Luz Lisboa, Lisbon, Portugal

**Keywords:** pregnancy, maternal immunity, B cells, T cells, immune reference ranges, flow cytometry

## Abstract

**Background:**

Pregnancy induces profound immunological adaptations necessary to support fetal development while preserving maternal health. However, the systemic dynamics of less-studied adaptive immune cell subsets across gestation remain incompletely understood.

**Objective:**

We have conducted a comprehensive longitudinal analysis of peripheral B and T cell populations in healthy pregnant women in order to identify trimester-specific immune changes and to establish reference intervals for clinical and research use.

**Methods:**

A total of 50 pregnant and 30 age-matched non-pregnant women were recruited in a prospective cohort study. Peripheral blood was collected at each trimester and analyzed by high-dimensional flow cytometry. We evaluated 74 lymphocyte subsets, including follicular and non-follicular CD4 and CD8 T cells, and functional markers CD69 and PD-L1, under basal and stimulated conditions.

**Results:**

Pregnancy was associated with decreased total B cell counts, particularly within transitional and anergic naïve subsets, and increased activated naïve and memory B cells. T cell activation progressively increased in CD4 and CD8 subsets, especially during late pregnancy. Notably, activated circulating follicular helper T cells (cTfh) were consistently reduced throughout gestation compared to controls, while CD69 and PD-L1 expressions on CD4 and CD8 T cells increased in the third trimester. Maternal factors, including age, parity, miscarriage history, and BMI, significantly influenced specific immune profiles. Reference intervals were established for key subsets, and deviations in women who experienced pregnancy complications suggest potential predictive value for future risk assessment.

**Conclusions:**

Our findings provide novel insights into the systemic immune adaptations that occur during pregnancy, particularly concerning follicular and non-follicular lymphocyte subsets. The proposed reference ranges proposed may serve as valuable tools for immunomonitoring and for identifying pregnancies at risk.

## Introduction

Pregnancy represents a unique immunological landscape characterized by complex and dynamic adaptations in the maternal immune system, essential to sustain maternal health and support fetal development. These adaptations involve intricate changes in both innate and adaptive immune responses to achieve an optimal balance between immune tolerance and defense mechanisms, including alterations in cytokine production, immune cell trafficking, and modulation of immune responses (1–3). The current paradigm supports a pro-inflammatory environment during early and late pregnancy, promoting blastocyst adhesion and tissue remodeling in the uterine wall during implantation, and facilitating labor-associated processes at term. In contrast, mid-pregnancy is regulated by anti-inflammatory mechanisms that are critical for fetal growth and development. Maintaining this delicate equilibrium is of paramount importance, as disruptions in these adaptations are increasingly associated with adverse pregnancy outcomes, including preterm labor, preeclampsia, and intrauterine growth restriction (4, 5). Furthermore, pregnant women may become more susceptible to infection during the anti-inflammatory phase, with potentially devastating consequences for both the mother and fetus (6, 7). Conversely, the course of pre-existing maternal autoimmune or allergic diseases may improve, worsen, or remain unchanged depending on the specific condition, reflecting the complexity of maternal immune adaptations (8, 9).

Studies investigating immune adaptations at the maternal-fetal interface have been particularly valuable in enhancing our understanding of the cellular mechanisms that control maternal immune tolerance (10–13). However, due to ethical considerations and strong evidence of both local and systemic effects of maternal-fetal cross-talk, research has increasingly focused on the systemic immune adaptations experienced by women during pregnancy (3, 14, 15). For instance, although the extent and dynamics of the Th1/Th2 balance remain context-dependent and not reproducible across studies (14), the shifts observed in these subsets are pivotal in orchestrating gestational immune adaptations and are modulated from early pregnancy onward (16). Similarly, the role of regulatory T cells (Tregs) is well established, although variations in their levels and functions remain an active area of research (17).

A reduction in circulating B cells and specific B-cell subsets during pregnancy has also been reported in several studies (15, 18–21), often accompanied by decreased responsiveness to mitogens and infectious agents (22), although data from early pregnancy remains limited. As for B cells with regulatory functions (Bregs), studies have proposed a significant role of these cells in maintaining fetal tolerance during the initial stages of pregnancy. Nevertheless, the absence of specific Breg markers and the phenotypic heterogeneity used to define these cells across studies hinder the ability to draw definitive conclusions (23–25).

Coordinated interactions among diverse immune cell types are essential for the development of protective immunity. Follicular helper T cells (Tfh) are cornerstone in this process as they deliver cognate and soluble signals that drive B cell proliferation, survival, affinity maturation, and differentiation into antibody-producing cells and long-lived memory B cells (26). While Tfh cells are classically found in secondary lymphoid organs, a smaller population of phenotypically similar cells can also be found in the periphery (circulating Tfh, cTfh). These cells are thought to represent a memory pool of Tfh cells in humans, expressing CXCR5 and sharing functional similarities to their lymphoid-resident counterparts (27). Given their role in regulating B cell responses, cTfh cells have been widely studied in autoimmune diseases (28). However, their role in pregnancy and the regulation of B cell responses in this context remains poorly understood. Further characterization of follicular CD4 and CD8 T cell subsets, using additional activation and maturation markers, is necessary to clarify the dynamics of follicular T cells during pregnancy.

Despite the growing body of data, much remains to be understood regarding changes in adaptive immune responses occurring throughout normal physiological pregnancy, particularly concerning less-studied subsets. Establishing appropriate reference ranges for these immunological fluctuations in pregnancy is crucial for distinguishing physiological adaptations from pathological deviations. A precise understanding of these changes is an essential first step toward identifying immunological deviations associated with pregnancy-related complications and enhancing maternal care and monitoring strategies.

To facilitate the establishment of such benchmarks and enable the early identification of immune-related pregnancy complications, in this study, we utilized flow cytometry to provide a comprehensive longitudinal analysis of less-studied immune cell subsets. Our focus was on B cells and both follicular and non-follicular activated T cell subsets across trimesters in healthy pregnant and non-pregnant women.

## Materials and methods

### Subjects and sample collection

In this prospective observational study, healthy 1^st^ trimester pregnant women (PW), aged 18 to 45 years, with naturally conceived childbearing potential, were recruited at Hospital da Luz Lisboa between June 2022 and July 2024 and followed until delivery. Gestational age at inclusion was estimated by ultrasound or last menstrual period. Only pregnant women with uncomplicated singleton pregnancies, who underwent at least two evaluations without any clinically related dropouts were included. Sequential age-matched non-pregnant healthy women (NPW) attending routine annual well-woman exams, were recruited as the comparator group.

Exclusion criteria for both groups included a history of diabetes, hypertension, autoimmune or active infectious diseases (including hepatitis, HIV, and CMV infection), or any other medical condition that could adversely affect the immune system or require immunomodulatory therapy. Additional exclusion criteria included a history of neoplasms and smoking within six months prior to peripheral blood sample collection. The use of any prenatal medication other than vitamins, folic acid, and iron supplements also led to exclusion.

All recruited pregnant women were required to have no history of relevant pregnancy-related complications (e.g., pre-term delivery, intrauterine growth restriction, neonatal ventilation, preeclampsia, recurrent pregnancy loss) and no ongoing pregnancy-related complications at the time of inclusion.

All participants signed a written informed consent prior to their inclusion in the study, which was approved by the Hospital da Luz (CES/49/2021/ME) and NOVA Medical School (167/2021/CEFCM) Ethics Committees and was conducted according to the recently updated Declaration of Helsinki (29). All laboratory and clinical data were anonymized before being analyzed.

### Study visits

Three visits were scheduled for the pregnant group, corresponding to the routine clinical assessments conducted during the 1^st^, 2^nd^, and 3^rd^ trimesters of pregnancy. Non-pregnant controls were sampled once, to provide baseline reference values for comparison. Peripheral blood samples were collected via venipuncture into EDTA-coated and heparinized tubes at each planned visit. At each visit, serum samples were also prepared, aliquoted, and stored at −80°C for potential future analyses of soluble mediators.

Baseline data collected at enrolment for all participants included demographics (age), anthropometrics (Body Mass Index, BMI), obstetric history, and systolic and diastolic blood pressure. Additional clinical parameters, including complete blood count, were retrieved from the hospital medical records on each visit.

For pregnant participants, data collected on the day of delivery included gestational age and mode of delivery. For newborns, recorded data included gender, weight, and 1-min and 5-min Apgar scores, a standardized assessment of neonatal status based on appearance, pulse, grimace, activity, and respiration, each graded 0–2 with a total score ranging from 0 to 10. Scores of 7–10 indicate normal neonatal adaptation.

### Immunophenotyping and flow cytometry

Full blood counts were determined by the hospital’s Laboratory Medicine Service using an automated hematology analyzer (XN-10™, Sysmex).

To ensure proper standardization between samples and minimize random processing mistakes, two pre-validated 8-color panels of fluorochrome-conjugated monoclonal antibodies (14 unique markers in total) were used for the identification and quantification of T and B cell subsets. Both panels were supplied in the format of lyophilized antibody mixtures in single tubes from ExBio (Praha, Czech Republic) and processed according to the manufacturer’s instructions. Briefly, 100 μL of EDTA-anticoagulated whole blood cells, pre-washed with 2 mL BD FACS Flow (BD Biosciences), were incubated with the monoclonal antibodies in the tubes for 20 min, followed by treatment with EXCELLYSE Easy solution for 10 min. Cells were then washed with 2 mL BD FACS Flow (BD Biosciences) for 5 min at 300xg and acquired on the flow cytometer within 30 min of protocol completion. All steps were performed at room temperature. A total of 74 lymphocytic subsets were characterized, represented as absolute counts or fractions of the respective parent populations. This included the characterization of B cell subsets as described by Sanz and collaborators (30).

Absolute T- and B-cell counts were calculated by multiplying the relative frequencies obtained by flow cytometry, following the gating strategy presented in **Supplementary Figures 1**-**3**), with the absolute lymphocyte counts provided by the patient’s full blood count results. All analyses were performed within 2 hours of blood collection and the lymphocyte percentages obtained from flow cytometry were consistent with those reported in the full blood count.

To evaluate stimulation-induced expression of CD69 and PD-L1 in the surface of B and T cells, heparinized whole blood samples were diluted 1:1 with Iscove’s Modified Dulbecco’s Medium (IMDM, Corning^®^) and incubated for 5 h at 37°C in a 5% CO_2_ atmosphere without (unstimulated) and with (stimulated) a combination of phorbol 12-myristate 13-acetate (PMA) (50 ng/mL, Sigma Aldrich), calcium ionophore (1 μg/mL, Sigma Aldrich), and lipopolysaccharide (LPS) (10 μg/mL, Sigma Aldrich) (31–33). Following incubation, red blood cells were lysed with BD PharmLyse™ Lysing Buffer (BD Biosciences), and samples were stained for surface markers (i.e., anti-CD45, anti-CD3, anti-CD8, anti-CD19, anti-CD69, and anti-PD-L1 antibodies), washed with 2 mL of BD FACS Flow (BD Biosciences) for 5 min at 300xg, and acquired on the cytometer within 30 min.

All acquisitions were performed in an 8-color BD FACS Canto II Flow Cytometer (BD Biosciences, San Jose, CA, USA) with BD FACS Diva software version 8.0.2 (BD Biosciences). Infinicyt™ 2.0 (Cytognos, SL. Salamanca, Spain) software was used for file quality control and to isolate the lymphocyte population. FCS files were then exported, and downstream analyses to determine subpopulation frequencies were performed in FlowJo™ v10.6.2 (BD Biosciences) software. Single-color compensation controls were generated using manufacturer-provided lyophilized cell-based tubes corresponding to each antibody in the cocktail. These were acquired once per lot, in accordance with the supplier’s instructions, to establish the compensation matrix.

Detailed information on all antibody panels and subsets analysis is presented in **Supplementary Tables 1** and **2**. Gating strategies are shown for a representative sample in **Supplementary Figures 1**–**4**.

### opt-SNE

Dimensionality reduction of multi-color flow cytometry data was performed using OMIQ software from Dotmatics (www.omiq.ai, www.dotmatics.com). FCS 3.0 files from all participants were imported, and up to 5780 CD19^+^ cells and 5000 CD3^+^ T cells from each sample were subsampled and concatenated for the analysis. The markers used for opt-SNE analysis (34) are described in the figure legends. The following default parameters were applied: Max Iterations = 1000, opt-SNE End = 5,000, Perplexity = 30, Theta = 0.5, Components = 2, and Verbosity = 25. The random seed was not user-selected but automatically generated by the software when the task was initiated (2136 for the B cell tube; 3556 for the T cell tube).

### Statistical analysis

Statistical analyses and visualizations were performed by using GraphPad Prism v10.4.0 for Windows (GraphPad Software, Boston, MA, USA, www.graphpad.com).

Categorical variables were presented as absolute frequencies and percentages, and associations between them were analyzed with the Chi-square or Fisher’s exact test. The normality of the data was assessed by visual inspection through QQ plots of the residuals and by using the D’Agostino–Pearson normality test when necessary.

Longitudinal changes in cellular populations were evaluated by mixed-effects model with Geisser–Greenhouse correction, followed by p-value correction with Tukey’s multiple comparisons test. The frequency and cellular concentration of each subset were modelled with timepoint (trimesters) as a fixed effect and patient as a random effect. Comparisons between the non-pregnant group and each time point were performed with Brown–Forsythe and Welch ANOVA or with Ordinary one-way ANOVA tests, as appropriate, followed by Dunnett’s T3 multiple comparisons test; otherwise, the non-parametric Kruskal–Walli’s test followed by Dunn’s multiple comparisons tests were used.

Two-way ANOVA was conducted, through a mixed-effects model, to evaluate the effects of trimester and clinical characteristics dichotomized into two levels (BMI, age, parity, and abortion history) on each dependent variable (each cellular subset). The interaction between these factors was also assessed, and multiple comparisons were performed with Tukey’s multiple comparisons test. The number of biological replicates (n) in each comparison can be found in the **Supplementary Material** associated with each result.

All the analyses described above were performed after the outlier exclusion of continuous variables using the ROUT method, as recommended by GraphPad, using a Q value of 0.1%. All tests were performed with and without outliers to verify their effect on p-value, and most of the results pointed out in the same direction (significant and non-significant values). We report here the results that do not include the outliers. All statistical tests performed were two-tailed.

Each test used is indicated in the respective figure and table legends. For all analyses, a p-value of less than 0.05 was considered significant: * p < 0.05, ** p < 0.01, *** p < 0.001, **** p < 0.0001.

As a measure of the magnitude of the difference, the effect size was calculated as described (35):

-For mixed-effects ANOVA: partial eta-squared (η_p_^2^) is small if ≥ 0.01 and < 0.06; medium if ≥ 0.06 and < 0.14 or large if ≥ 0.14;

-For Unpaired t test/Dunnett’s test: Cohen’s *d* (*d*) is small if <0.3; medium if ≥0.3 and <0.8 or large if ≥0.8.

The effect size values are reported in figures and/or **Supplementary Material** and are labelled as ^+^ for small, ^++^ for medium, and ^+++^ for large effect sizes according to these values.

For reference interval calculation, we followed the CLSI EP28-A3C guidelines (36). Briefly, raw data in each subset were tested for normality using histograms and the Shapiro-Wilk test. Possible outliers were identified using the Dixon-Reed rule and removed before further analysis. For skewed distributions, a log transformation was tested to improve normality and assess whether it enhanced reference interval estimation. Since the sample size is < 120 participants, the robust method was used to calculate lower and upper limits, with 90% confidence intervals estimated using the bootstrapping method with 10000 iterations and a 978 random number seed. If the robust method produced upper or lower limits that exceeded considerably the observed minimum or maximum values, the reference range was adjusted using non-parametric percentiles (2.5th and 97.5^th^, respectively), to avoid creating a reference range that extends beyond real-world data. All analyses were performed using MedCalc^®^ Statistical Software version 23.1.7 (MedCalc Software Ltd, Ostend, Belgium; https://www.medcalc.org; 2025).

## Results

### Study population

**Figure 1:**
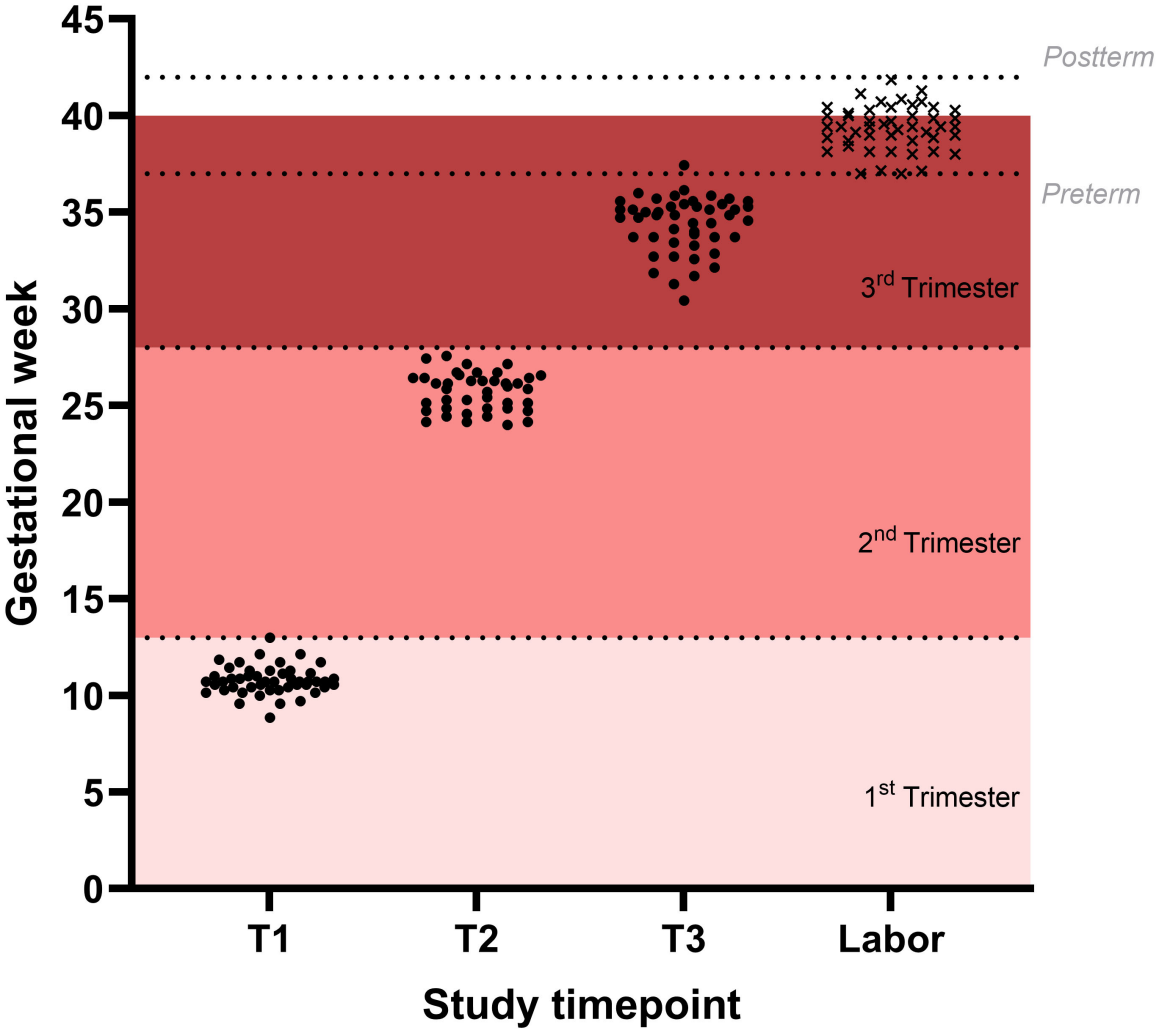
Distribution of sampled time points in the study population. Sampled time points are shown for the three visits during gestation (black dots) and time of parturition (black x).

A total of 60 pregnant and 30 non-pregnant women were recruited for this study. In the pregnant group, 10 participants were excluded, 4 due to pregnancy complications and 6 due to non-clinical loss to follow up, resulting in a final cohort of 50 participants. Baseline characteristics of both pregnant and non-pregnant women are presented in **Table 1**, and the distribution of sampling time points is summarized in **Figure 1**. Of the 150 expected samples from the pregnant group, 7 samples could not be collected due to non-clinical reasons, such as missed scheduled appointments without prior notice or transfer of care to another hospital.

**Table 1:** Demographic and clinical characteristics of included groups.

Characteristics	PW (n=50)	NPW (n=30)	P-value
Age*, years ≤ 33 > 33	33.2 (4.0) 27 (54%) 23 (46%)	32.2 (5.5) 21 (70%) 9 (30%)	**0.390^a^** **0.238^c^**
BMI*, kg/m^2^ ≥18.5 and <25 ≥25 and <30 ≥30	24.8 (4.8) 32 (64%) 11 (22%) 7 (14%)	23.4 (3.3) 21 (70%) 7 (23%) 2 (7%)	**0.142^a^** **0.603^b^**
Systolic pressure*, mmHg	118.0 (11.0)	117.7 (10.3)	**0.998^a^**
Diastolic pressure*, mmHg	71.8 (7.1)	72.1 (8.6)	**0.826^a^**
Parity, range Nulliparous Primiparous Multiparous	0-3 29 (58%) 14 (28%) 7 (14%)	0-4 11 (37%) 10 (33%) 9 (30%)	**0.117^b^**
Miscarriage history**, range <1 ≥1 and ≤2 >2	0-3 31 (62%) 17 (34%) 2 (4%)	0-2 26 (87%) 4 (13%) 0	**0.038^c^**
Gestational age at delivery, weeks	39.4 (1.1)	**-**	**-**
Type of delivery, n (%) Vaginal Caesarean Elective Intrapartum	29 (60%) 19 (40%) 3 (16%) 16 (84%)	**-**	**-**
Newborn’s birth weight, grams	3216 (375)	**-**	**-**
Fetal sex, female, n (%)	21 (44%)	**-**	**-**
APGAR score, median [range] 1-min 5-min	9.5 [6 - 10] 10 [9 - 10]	**-**	**-**

Data are listed as mean (standard deviation) or number n (%), otherwise indicated. *Data collected at study inclusion. **Non-consecutive episodes. ^a^Unpaired t-test, ^b^Qui-square test, ^c^Fisher’s exact test. APGAR normal range: 7–10. PW, pregnant women; NPW, non-pregnant women; BMI, body mass index.

The mean age and BMI were similar between groups. In the pregnant group, 46% of the women were older than 33 years, whereas age distribution in the non-pregnant group was more evenly balanced, with approximately one-third in each age range. Regarding BMI, both groups primarily consisted of women with normal weight (18.5 to 24.9 kg/m^2^). Most women in both groups were nulliparous, however, the pregnant group had a higher frequency of women with one or more non-consecutive miscarriages. No additional significant differences were observed between the groups regarding age, BMI, or parity distribution.

The mean gestational age was 10^+5^ weeks at the first visit (T1), 25^+5^ weeks at the second visit (T2), and 34^+3^ weeks at the third visit (T3). All women successfully delivered, with a mean gestational age at delivery of 39^+3^ weeks. Regarding delivery timing, no cases of preterm (<37 weeks) or post-term (≥42 weeks) labor were recorded, and 65% of deliveries occurred at full term (≥39 and <41 weeks). Vaginal delivery was the most common mode of parturition (60%). The mean weight of the newborn was 3216 grams, and 44% were female.

### Leucocyte changes across trimesters

**Figure 2:**
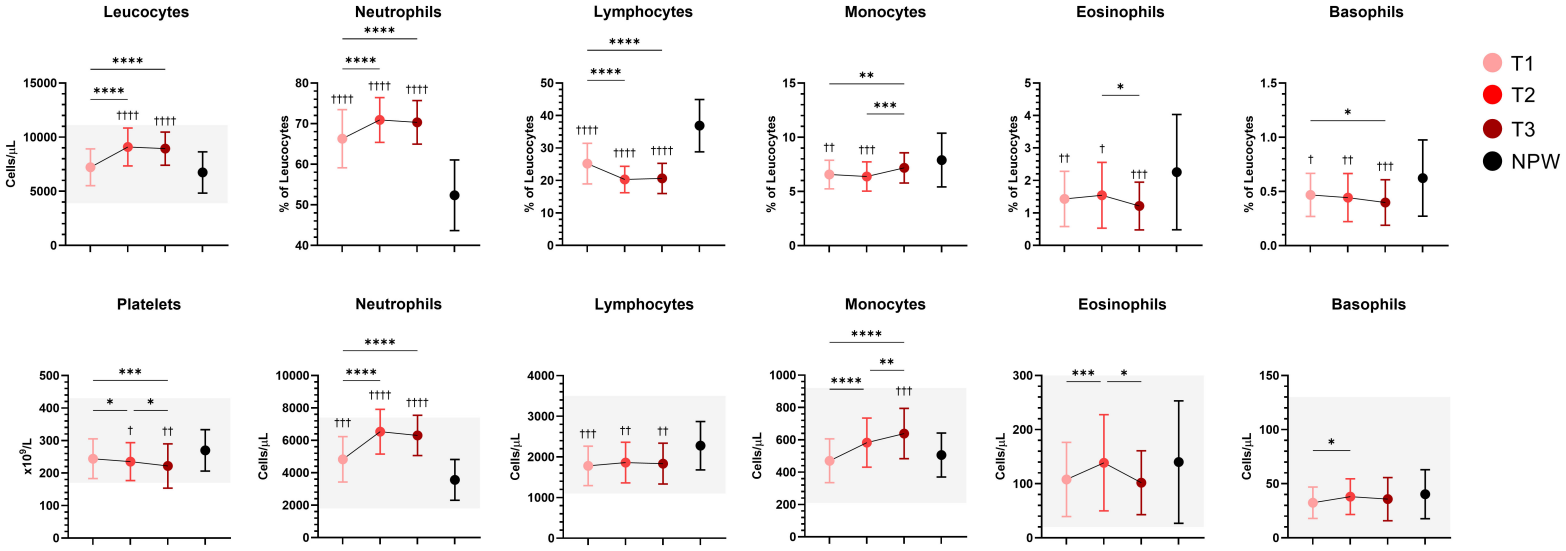
Longitudinal changes in peripheral blood cell populations during pregnancy. Percentages and/or absolute counts of main leucocyte immune populations and platelets across pregnancy and in non-pregnant women are presented. Mean with standard deviation of the indicated population across groups are indicated, and normal ranges (when available) are represented with shaded areas. Statistical significance between trimesters was determined with mixed-effects analysis followed by Tukey’s multiple comparisons test, and are indicated as: *p < 0.05, **p < 0.01, ***p < 0.001, ****p < 0.0001. Comparisons between pregnant and non-pregnant groups were performed with ordinary One-way ANOVA followed by Dunnett’s multiple comparison test, and are indicated as ^†^ *p* < 0.05, ^††^p < 0.01, ^†††^p< 0.001, ^††††^ *p* < 0.0001.

Changes in circulating immune cell composition during pregnancy have been widely documented, revealing dynamic shifts across different leukocyte subsets. In line with this, we observed several notable trends among major leucocyte subsets, particularly neutrophils and monocytes (**Figure 2**). Neutrophils and monocyte count increased significantly from the first trimester of pregnancy onwards, whereas lymphocyte levels remained stable and consistently lower than those observed in the non-pregnant group. Notably, significant differences between pregnant and non-pregnant women were observed across several of the studied cell populations. However, overall, all leukocyte subsets in pregnant women remained within the reference ranges used for healthy adult women. Detailed erythrogram and leucogram results are provided in **Supplementary Table 3** and **Supplementary Figure 5**.

### T and B lymphocyte subsets across trimesters

Major adaptations occur in the maternal immune system to ensure the success of pregnancy, and dysregulation of these mechanisms is increasingly implicated in the pathogenesis of several pregnancy-related complications (4). However, a clear understanding is still lacking regarding the mechanisms by which these immunological changes, particularly those concerning B and T cell subsets, occur and how they may influence the susceptibility to infection and the progression of immune-mediated diseases during pregnancy.

**Figure 3:**
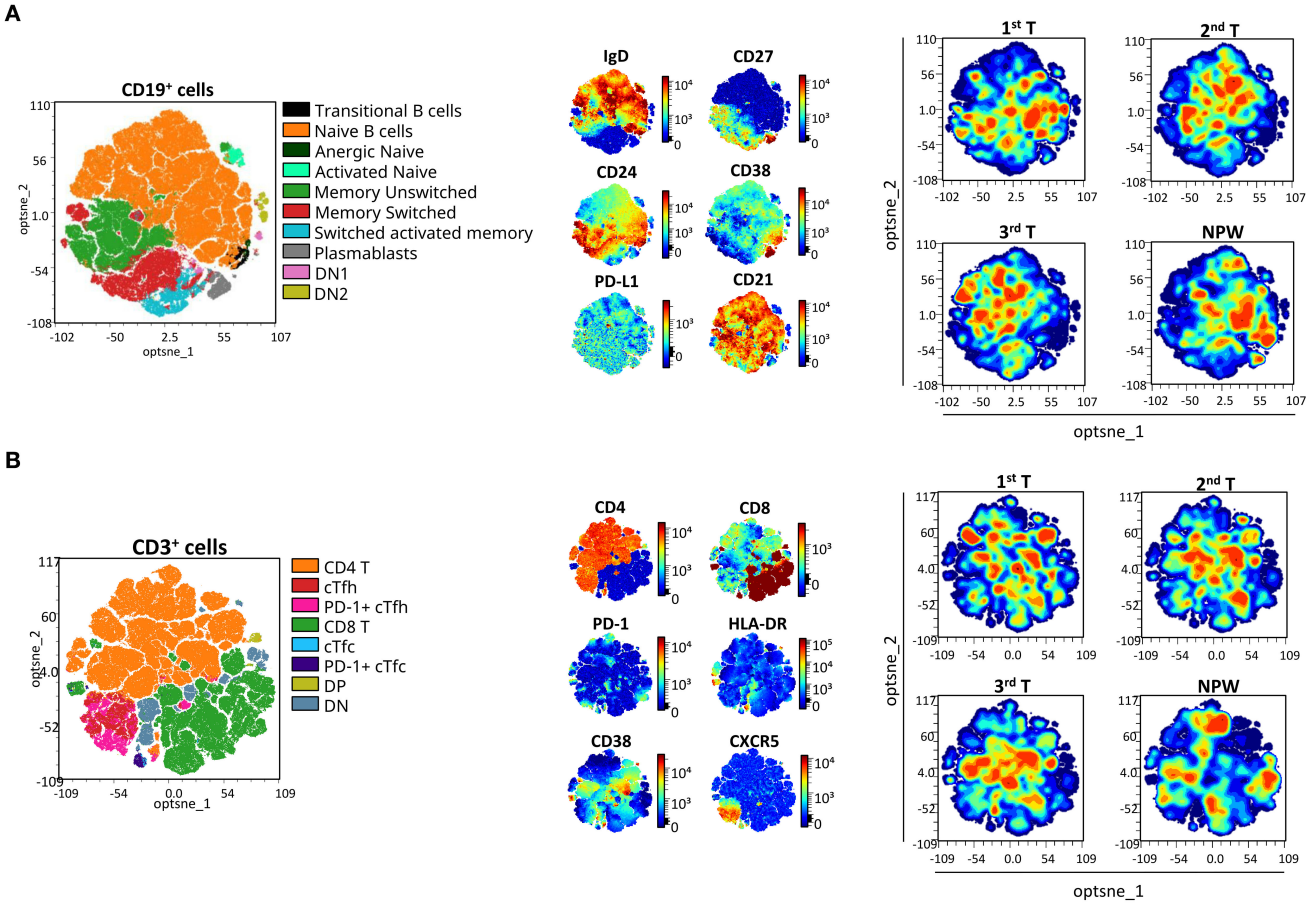
opt-SNE mapping of B and T cells from pregnant and non-pregnant groups. opt-SNE plots were generated from flow cytometry data for **(A)** CD19^+^ B cells and **(B)** CD3^+^ T cells, using the indicated markers. Manually gated populations are shown on the left for each cell type, colored by subset identity. Marker expression patterns are visualized in the middle panels, and density plots for each pregnant timepoint (1^st^, 2^nd^, and 3^rd^ trimester) and non-pregnant women (NPW) are shown on the right to illustrate group-level distributions. For T-cell subsets, cTfh were defined as CXCR5^+^CD4^+^ T cells, with PD-1 expression used to identify activated cTfh subsets. cTfc were defined as CXCR5^+^CD8^+^ T cells, with PD-1 expression used to identify activated cTfc subsets. DP, double positive; DN, double negative; cTfh, circulating T follicular helper; and cTfc, circulating T follicular cytotoxic cells.

Among the 74 cellular subsets characterized, differences in the frequency of B and T cell subsets were observed across all three trimesters of pregnancy, and in comparison with the non-pregnancy state. **Figure 3** illustrates, in an unsupervised manner, the overall distribution of B and T cell populations and the expression of analyzed markers across groups using opt-tSNE.

**Figure 4:**
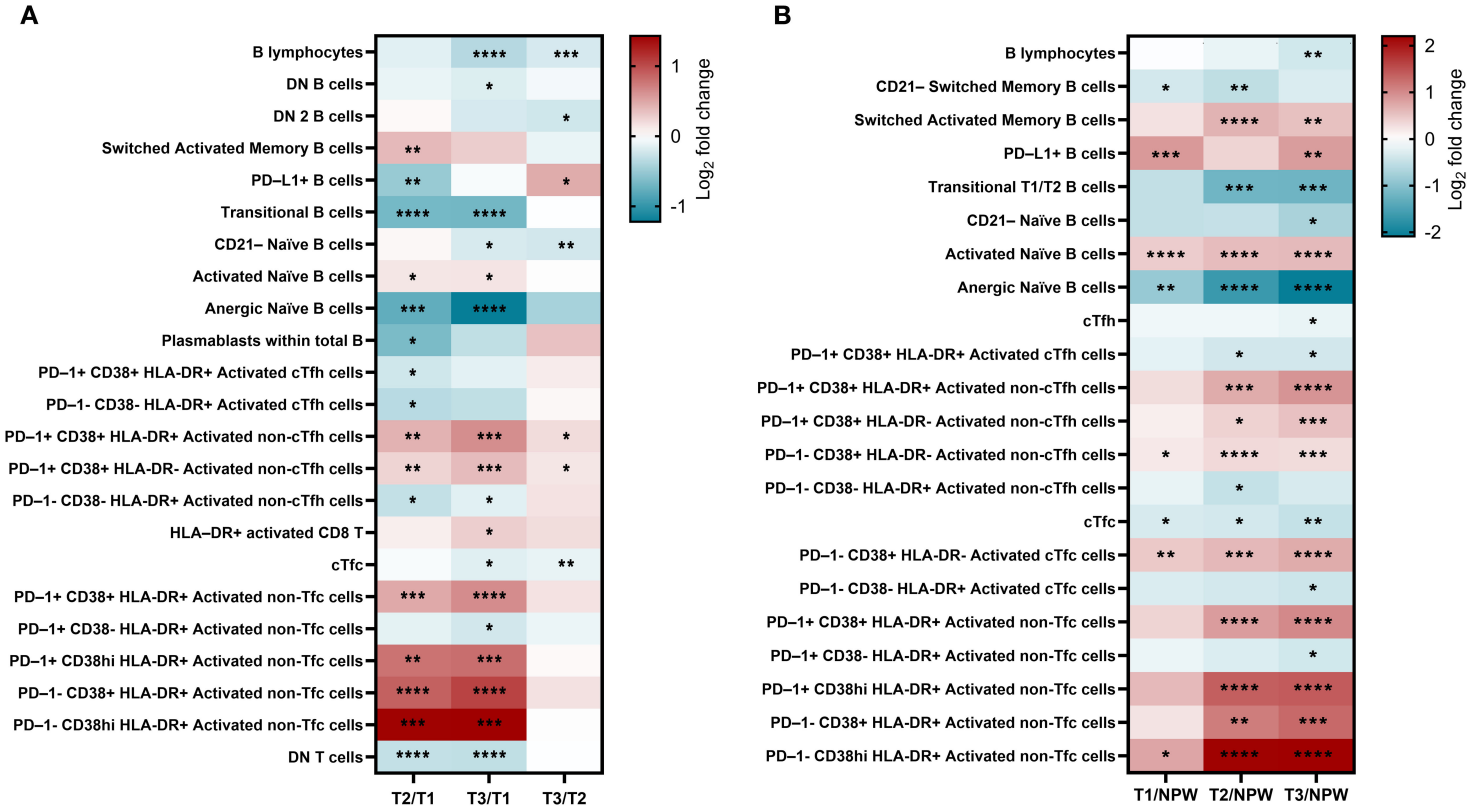
Changes in frequency of peripheral blood lymphocytic populations in pregnant and non-pregnant groups. **(A)** Longitudinal comparisons between the 1^st^ (T1), 2^nd^ (T2), and 3^rd^ (T3) trimester study visits. **(B)** Comparisons between non-pregnant women (NPW) and each trimester of pregnant group. Only subsets of immune cell populations with a fold change greater than 1.1 or less than 0.9 and significant differences for at least one comparison are represented. Statistical significance was calculated using Tukey’s **(A)** or Dunnett’s **(B)** multiple comparisons test and are denoted as follows: *p < 0.05, **p < 0.01, ***p < 0.001, and ****p < 0.0001. The color bar represents fold change on a Log_2_ scale of each subset’s percentage relative to its parent population (0 = no change). Detailed information is available in **Supplementary Table 3**.

In detail, 24 subsets showed significant changes across pregnancy timepoints (**Figure 4A**), and 23 differed significantly when compared to the non-pregnant group (**Figure 4B**). Inclusion criteria were defined as a mean fold change ≥ 1.1 (10% increase from baseline) or ≤ 0.9, and p < 0.05. Our findings highlight several pregnancy-specific immune variations that were consistent across trimesters but also distinct from the non-pregnant state (**Figure 4**). Specifically, total B cells, transitional B cells, and anergic naïve B cells demonstrated a progressive decline throughout pregnancy, with the most significant reductions occurring in late pregnancy. These decreases were evident in both within-pregnancy pregnancy vs non-pregnant comparisons. In contrast, activated naïve and switched memory B cells increased from early to late pregnancy, indicating a shift toward heightened B cell activation. These activated subsets were also significantly elevated compared to non-pregnant women.

PD-L1^+^ B cells followed a non-linear trajectory, decreasing from early to mid-pregnancy and then rising in late pregnancy. Notably, levels were consistently higher in early and late pregnancy relative to the non-pregnant group. Some changes, such as those observed in plasmablasts, were only detected within-pregnancy comparisons and not when compared to the non-pregnant women. Collectively, these results underscore a finely regulated, pregnancy-specific modulation of B-cell activity that balances immune tolerance and adaptation to support fetal development.

Dynamic changes were also observed in T cell populations, with significant increases in circulating non-follicular helper (non-cTfh) and cytotoxic (non-cTfc) activated subsets, particularly in late pregnancy, suggesting robust pregnancy-induced activation. CD8 T cell activation was especially prominent in mid and late pregnancy, both in within-pregnancy comparisons and relative to non-pregnant women, underscoring their critical role in immune adaptation during these stages.

Notably, some changes were more apparent when comparing pregnant to non-pregnant women. For instance, the activated PD-1^+^ CD38^+^ HLA-DR^+^ cTfh subset was significantly decreased in pregnant women compared to non-pregnant controls but showed no major variation across trimesters. This suggests that cTfh suppression reflects a baseline immunological shift associated with pregnancy, rather than trimester-specific modulation. Conversely, double-negative (DN) T cells, were consistently reduced throughout pregnancy and in comparison to non-pregnant women, despite the stable levels of total CD4 and CD8 T cells. These observations suggest tightly regulated immune adaptations, particularly within the CD8 T cell compartment, aimed at preserving the immune balance essential for both maternal and fetal well-being during pregnancy. Detailed results for all subsets are presented in **Supplementary Table 3**.

In addition to proportional changes observed in B and T cell subsets, we observed a significant decrease in the absolute counts of total B and T cells during pregnancy, accompanied by a general reduction across their respective subsets (**Supplementary Figure 6**). Despite this overall decline in lymphocyte counts, the levels of distinct activated CD4 and CD8 T cell subsets were notably higher in late pregnancy compared to non-pregnant women. This was particularly evident in PD-1^+^ CD38^+^ HLA-DR^+^ non-cTfh cells, PD-1^+^ CD38^+^ HLA-DR^+^ non-Tfc cells, and PD-1^-^ CD38^+^ HLA-DR^+^ non-Tfc cells. Furthermore, a consistent increase in the absolute counts of activated subsets of CD4 and CD8 T cells was observed, aligning with the trends seen in their respective frequencies within the maternal immune compartment. Detailed results for these findings are presented in **Supplementary Table 4**.

**Figure 5:**
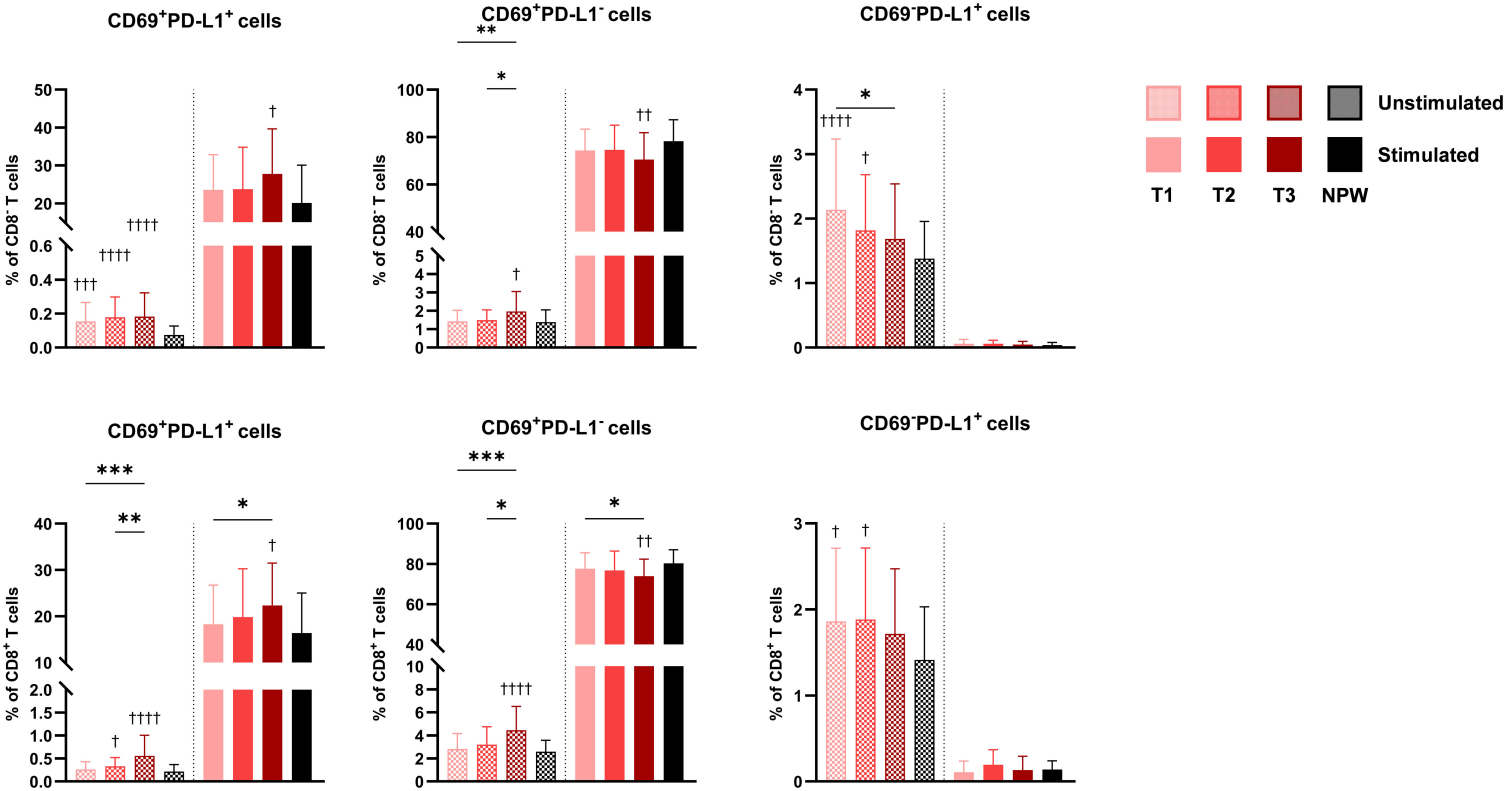
CD69 and PD-L1 expressing cells in pregnant and non-pregnant women. Percentages of CD69^+^PD-L1^+^, CD69^+^PD-L1^+^, and CD69^-^PD-L1^+^ in total CD4 (top row) and CD8 T cells (bottom row) across pregnancy (T1-T2-T3) and between non-pregnant women (NPW). Data are presented for both unstimulated (hatched bars) and stimulated (solid bars) conditions. Each bar displays the mean and standard deviation of the indicated population across groups. Stimulated bars indicate baseline-corrected to each participant’s matched unstimulated condition (stimulated − unstimulated). Statistical significance between trimesters was determined with mixed-effects analysis, followed by Tukey’s multiple comparisons test, and is indicated as: *p < 0.05, **p < 0.01, ***p < 0.001. Comparisons between pregnant and non-pregnant groups were performed with ordinary One-way ANOVA followed by Dunnett’s multiple comparison test, and are indicated as ^†^ *p* < 0.05, ^††^p < 0.01, ^†††^p < 0.001, ^††††^ *p* < 0.0001.

In line with the increased proportion of activated cells observed in late pregnancy, we found significantly higher basal frequencies of CD69-expressing CD8^-^ and CD8^+^ T cells, particularly in the third trimester (**Figure 5**), whereas B cells did not show significant differences. Additionally, a higher proportion of CD8^-^ and CD8^+^ T cells co-expressing CD69 and PD-L1 markers was observed in pregnant women compared to non-pregnant women, with the most pronounced differences in the third trimester. In contrast, cells expressing PD-L1 alone were more frequent among CD8^-^ and CD8^+^ T cells in early pregnancy but less prevalent in non-pregnant women.

Following stimulation, pregnancy was not associated with major changes in immune cell activation capacity based on the markers analyzed. However, an increased proportion of CD69^+^PD-L1^+^ cells was observed in the third trimester after stimulation. These findings suggest that pregnancy is associated with a modest increase in the baseline activation of peripheral CD8^-^ and CD8^+^ T cells, peaking in late pregnancy, without a substantial impact on their capacity to respond to stimulation.

Detailed results are provided in **Supplementary Table 5**.

### Associations with clinical and demographic characteristics

Recognizing that variability in clinical and demographic characteristics could influence the immune profiles observed in the pregnant group, we further stratified this cohort based on key variables. Specifically, we examined parity (nulliparous vs. multiparous), maternal age (<33 years vs. ≥33 years), history of prior miscarriage (< 1 vs. ≥ 1 miscarriage), and BMI (<25 vs. ≥25 kg/m²). Significant findings are summarized in **Figure 5**, with additional statistical details provided in **Supplementary Table 6**. To ensure relevance and minimize confounding, we focused only on immune subsets for which corresponding non-pregnant subgroups, stratified by the same criteria, showed no significant differences.

**Figure 6:**
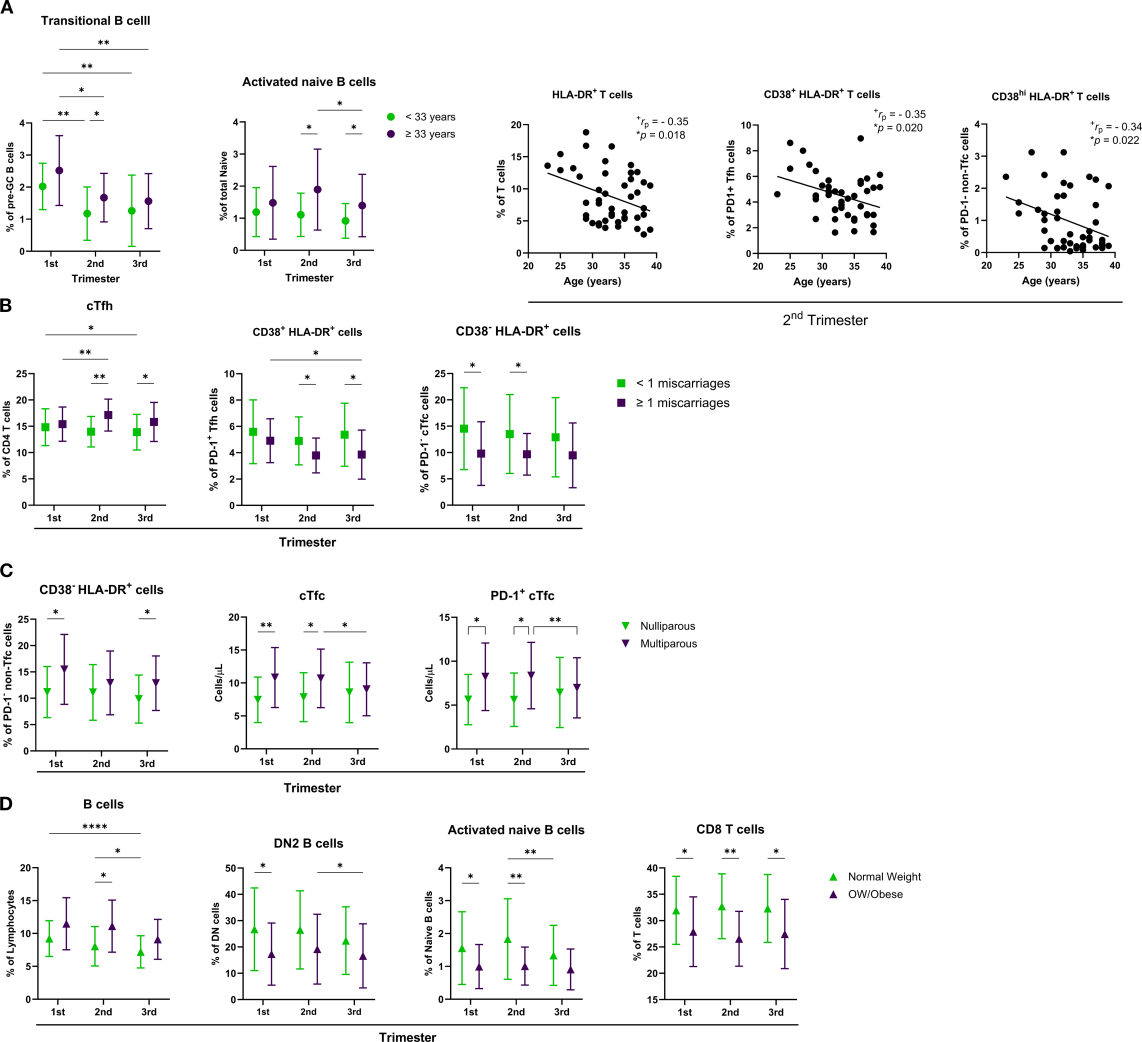
Influence of maternal characteristics on immune cell populations across pregnancy. Distribution of various immune cell subsets across the first, second, and third trimesters, stratified by **(A)** maternal age (<33 vs. ≥33 years), **(B)** history of miscarriage (<1 vs. ≥1), **(C)** parity (nulliparous vs. multiparous), and **D**) body weight (normal weight vs. overweight/obese). Correlation plots show the association between maternal age and the percentages of HLA-DR^+^ T cells, CD38^+^ HLA-DR^+^ PD-1^+^ Tfh cells, and CD38^hi^ HLA-DR^+^ PD-1^-^ non-Tfc cells during the 2^nd^ trimester. Data are presented as mean ± SD. Statistical significance is indicated by *p < 0.05, **p < 0.01, ***p < 0.001, and ****p < 0.0001, and was determined by Tukey’s multiple comparisons test or by Pearson Correlation. Effect size measures ^+^small were determined by *r* – correlation coefficient *r*.

Maternal age exhibited a small but significant effect on the levels of transitional B cells and activated naïve B cells, which were higher in older individuals (**Figure 6A**). Additionally, negative correlations between maternal age and T cell subsets were observed during mid-pregnancy, particularly in HLA-DR^+^ T cells, PD-1^+^ CD38^+^ HLA-DR^+^ activated cTfh, and PD-1- CD38^hi^ HLA-DR^+^ activated non-cTfc cells. Maternal age was not associated with changes in the absolute counts of the immune populations analyzed.

Among the pregnant participants, 19 women had a history of at least one prior miscarriage. These women were, on average, 3.4 years older than those without such a history. When stratified by miscarriage history, a significant difference in the percentage of cTfh cells was observed, along with a significant interaction between trimester and miscarriage history (**Figure 6B**). Prior miscarriage history was also associated with increased levels of activated PD-1^+^ CD38^+^ HLA-DR^+^ cTfh cells and activated PD-1^-^ CD38^-^ HLA-DR^+^ cTfc cells. No significant differences were found in B cell subsets or absolute counts of the analyzed populations based on miscarriage history.

In our cohort, 21 pregnant women were multiparous and were, on average, 2.5 years older than nulliparous women. Multiparous participants showed increased frequencies of activated PD-1^+^ CD38^+^ HLA-DR^+^ non-Tfc cells and higher counts of cTfc and PD-1^+^ cTfc cells during early and mid-pregnancy (**Figure 6C**).

When comparing low-normal weight (BMI < 25 kg/m^2^) to overweight or obese (BMI > 25 kg/m^2^) pregnant women, several significant effects were observed (**Figure 6D**). Overweight and obese women exhibited higher levels of total B cells during the second trimester, alongside decreased levels of DN2 B cells and activated naïve B cells during early to mid-pregnancy. Lower percentages of CD8 T cells were consistently observed throughout pregnancy in this group. No significant differences in absolute cell counts were detected between the BMI groups.

### Establishing reference values for cellular subsets: calculation and clinical significance

**Figure 7:**
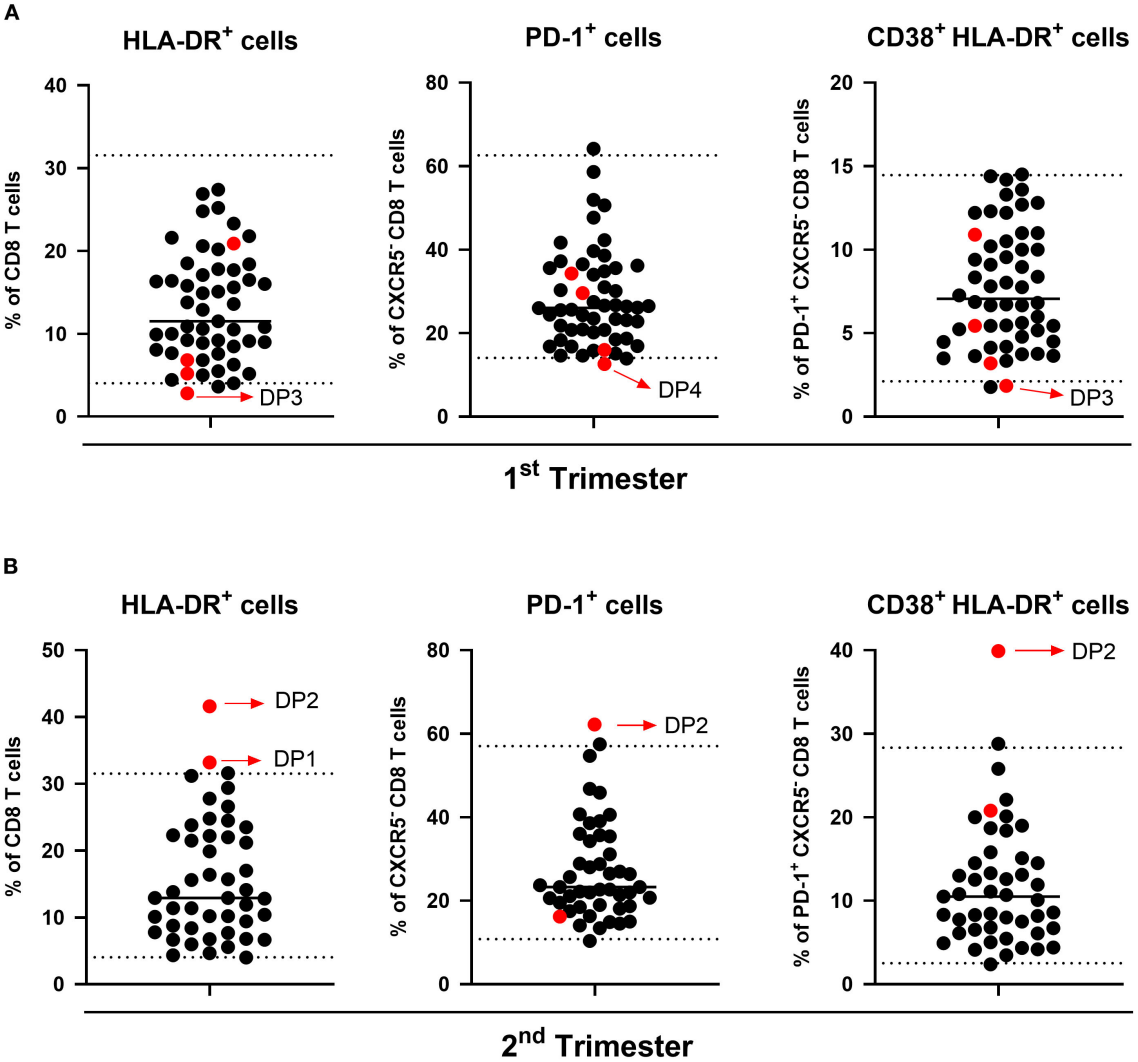
Distribution of immune cell populations in clinical dropout participants. Scatter plots show the percentage of HLA-DR^+^ CD8 T cells (left), PD-1^+^ non-Tfc cells (middle), and CD38^+^ HLA-DR^+^ PD-1^+^ non-Tfc cells (right) in the **(A)** 1^st^ and **(B)** 2^nd^ trimesters. Each black dot represents participants with healthy pregnancies, used to calculate reference intervals. Red dots indicate clinical dropouts (DP) in this study. The dotted lines represent reference thresholds corresponding to the 2.5^th^ and 97.5^th^ percentiles.

Given the observed changes in B and T cellular subsets across trimesters and the differences compared to the non-pregnant group, we calculated reference intervals for these subsets in each trimester of gestation, as presented in **Supplementary Tables 7** and **8**. To explore their potential clinical significance, we compared the reference values with data from four participants who discontinued the study due to adverse outcomes (**Figure 7**). Three participants were transferred to a tertiary center because of pregnancy complications: oligohydramnios (DP1), premature rupture of membranes (DP2), and vasa previa (DP3). One participant had a missed miscarriage in the first trimester (DP4). In all four cases, at least one cellular subset fell outside the established reference range in the timepoint preceding the adverse outcome. A total of seven subsets showed notable deviations, primarily with values exceeding the upper reference limit. Among these, the CD8 T cell subsets were the most frequently affected, specifically, activated HLA-DR^+^ CD8 T cells, PD-1^+^ non-cTfc cells, and PD-1^+^ CD38^+^ HLA-DR^+^ non-cTfc subsets, suggesting a potential link between dysregulation in the populations and negative clinical outcomes. Due to the reduced sample size, no statistical analysis was possible.

## Discussion

Advances in cytomics and single-cell transcriptomics have significantly enhanced the ability to capture the complexities of systemic immunological adaptations during pregnancy (15, 37, 38). However, there remains a critical need to monitor immune dynamics longitudinally within the same individual using accessible biological compartments. Such approaches must yield rapid, interpretable, and clinically actionable insights, capabilities well supported by flow cytometry-based routine immunophenotyping.

This study presents a high-dimensional analysis characterizing the phenotypes and functions of peripheral immune T and B cell populations in maternal peripheral blood samples assessed longitudinally throughout normal pregnancy and compared to non-pregnant women. We validate previously reported immunological changes during pregnancy and provide novel insights into alterations in maternal peripheral immunity. Additionally, we propose preliminary reference intervals for key immune cell subsets, offering a potential framework for monitoring pregnancy progression and identifying early deviations that may precede pregnancy complications.

Significant changes in leukocyte subpopulations were observed throughout pregnancy, with a progressive increase in neutrophil and monocyte concentrations beginning in the first trimester, while lymphocyte levels remained stable and consistently lower than those in non-pregnant women. These findings are consistent with the well-established phenomenon of physiological leukocytosis during pregnancy (14, 39). The early post-implantation of the first trimester is characterized by sustained levels of inflammatory cytokines and growth factors, such as Granulocyte and Granulocyte-Macrophage Colony Stimulating Factors (G-CSF and GM-CSF, respectively), which drive neutrophil and monocyte production in the bone marrow (3, 40, 41). Additionally, pregnancy-associated hormones such as estrogen and progesterone may also contribute to these alterations by extending the neutrophil lifespan through inhibition of apoptosis (42, 43). This innate-driven inflammation plays a key role in supporting wound healing, post-implantation recovery, placentation, and uterine remodeling (44). Importantly, although peripheral neutrophils and monocytes increase dramatically in numbers, they are functionally reprogrammed to promote immune tolerance (45, 46). Neutrophils, while more abundant, show reduced pro-inflammatory activity, produce immunoregulatory mediators that inhibit T cell responses, while releasing pro-angiogenic factors at the decidua to support placental development. Pregnancy hormones and placental factors further skew neutrophils toward an anti-inflammatory state and enable crosstalk with adaptive immunity, promoting the induction of Treg cells secreting IL-10 and VEGF (47). Circulating monocytes are similarly reprogrammed by placental signals (46). First-trimester placental exosomes convert monocytes into an M2-like state with high PD-L1 expression, leading to suppressed CD4^+^/CD8^+^ T-cell proliferation and expanded Tregs (48). In parallel, trophoblast microparticles elicit moderate monocyte secretion of TNF-α, IL-6, and IL-8, contributing to a controlled inflammatory milieu (46). Throughout gestation, monocytes undergo epigenetic remodeling and subset shifts, more significant in intermediate CD14^+^CD16^+^ cells, that heighten their activation potential by term (46). Together, these innate adaptations help establish maternal-fetal tolerance, while their dysregulation is linked to complications such as preeclampsia and miscarriage.

Observed alterations in erythrogram components and the progressive decline in platelet counts are well-documented physiological adaptations in pregnancy, often attributed to increased platelet turnover and hemodilution (49).

Of the 74 lymphocyte subsets analyzed in this study, 24 subsets exhibited significant changes across pregnancy timepoints, while 23 subsets differed significantly when compared to the non-pregnant group. Peripheral blood B cell levels varied throughout normal pregnancy and differed from those in non-pregnant women. A notable decrease in total circulating B cells was particularly evident during the third trimester, a finding that has been widely reported in previous studies (19, 50, 51).

Previous studies have linked these changes in the B cell compartment to endocrine factors such as human chorionic gonadotropin (hCG), which modifies B cell differentiation and function (52, 53). Muzzio et al. also demonstrated that B lymphopoiesis in mice decreases in late pregnancy, coinciding with elevated estradiol levels (54). Another likely mechanism contributing to reduced peripheral B cell levels is their migration and retention at the maternal-fetal interface, a hypothesis supported by studies highlighting the critical role of B cells in this region (55).

Moreover, most B cell subsets exhibit decreased absolute counts during pregnancy (14), with transitional B cells showing particularly pronounced reductions in late pregnancy (21, 51, 56). However, no significant differences were observed in the distribution of naïve and memory B cell subsets across study visits. Nonetheless, variations in the proportions of major B cell subsets have been inconsistently reported in studies, likely due to distinct phenotypic and methodological approaches.

Plasmablasts decreased from the first to the second trimester but did not differ significantly from levels in non-pregnant women, contrary to previous reports (15, 51). However, naïve and memory B cell populations showed distinct activation dynamics: anergic naïve B cell proportions showed a continuous decline throughout pregnancy, while activated naïve and switched memory B cells progressively increased from early to late gestation. A study by Demery-Poulos et al. found a greater proportion of B cells in pregnant women displaying memory-like and activated phenotypes (57) whereas Apps et al. reported opposite trends in these subsets (21). Additionally, a recent study reported downregulation of genes associated with signal transduction pathways, B cell activation, and immune responses in B cells (memory, naïve, and plasmablasts) throughout gestation and in comparison to the non-pregnant state (15). Similar findings were observed in total splenic B cells from pregnant and non-pregnant mice (58). Conversely, Demery-Poulos et al. found a significantly greater fold change in B cell activation following anti-human IgM/IgG stimulation in cells isolated from pregnant compared to non-pregnant women, suggesting pregnancy-enhanced circulating B cell responsiveness (57). Supporting this idea, higher levels of serum B-cell activating factor (BAFF) have been reported in pregnant women compared to non-pregnant, suggesting that BAFF may prime B cells and contribute to the pregnancy-specific increase in activation observed in these cells (59). In contrast to these studies, we found no significant changes in the B-cell activation status, as assessed by CD69 expression, at any gestational age, either at baseline or after stimulation. Still, our assessment only focused on the total B cell compartment and did not discriminate between different subsets within it.

PD-L1 expression on B cells defines a mechanism for potent suppression of humoral immunity by attenuating T cell activation and antibody production (60). In our study, PD-L1^+^ B cell levels decreased from early to mid-pregnancy and rose again in late pregnancy, with levels remaining elevated during the early and late stages compared to non-pregnant individuals. These dynamic fluctuations are consistent with existing literature on immune modulation during pregnancy and suggest a temporally regulated role for PD-L1^+^ B cells in maintaining immune balance across gestation (61, 62). For instance, a study by Mach et al. investigated soluble PD-L1 (sPD-L1) serum levels in uncomplicated pregnancies and found a significant increase throughout gestation, with the highest concentrations in the third trimester (63). Similar results were reported by Hedman et al. (64) and Okuyama et al. (65). Since PD-L1 is highly expressed by syncytiotrophoblast and its expression increases with gestational age, the placenta may serve as an important source of circulating PD-L1 (66). While these studies primarily focused on sPD-L1, they support the concept of dynamic PD-L1 expression during pregnancy, which may reflect changes in basal PD-L1^+^ B cell populations.

A recent study reported negative correlations between PD-L1^+^ B cells and various subsets of Tregs, i.e., subsets expressing markers that influence immunosuppressive function in early pregnant women (62). In fact, elevated PD-L1 expression in regulatory B cells may suppress inflammation by interacting with PD-1 expressed on T cells and Treg cells, thus delivering inhibitory signals that mediate immune suppression (67, 68). The increased levels observed during early pregnancy may facilitate the expansion of Tregs and a shift toward an immunotolerance state during mid-pregnancy. In the third trimester, increased PD-L1 expression on B cells can modulate immune responses, to ensure they do not adversely affect the fetus while facilitating the onset of labor. Interestingly, a study demonstrated that CpG-induced PD-L1 expression on human B cells can suppress Th2 cytokine production by CD4 T cells stimulated with pollen antigens, while increasing INF-γ and IL-12 secretion (69). In our study, the lower levels of PD-L1^+^ B cells observed during the second trimester might be related to the well-documented immune shift towards a Th2-dominant state during mid-pregnancy (14). Conversely, the increased levels of PD-L1+ B cells in late pregnancy may afterwards facilitate Th1 cytokine responses in this later stage.

However, it is important to note that immune associations observed in peripheral blood and circulating subsets may not directly reflect those in decidual tissues. Further research is, thus, needed to better characterize the PD-L1 expression on B cells throughout the full course of pregnancy, also locally, given the importance of the PD-1/PD-L1 axis at the maternal-fetal interface (61).

Previous evidence suggests that human circulating transitional B cells are enriched in regulatory B cells (70), and somehow exhibit regulatory properties (71). In our study, we observed a decline in CD24hiCD38^hi^ T1/T2 transitional B cells beginning in the first trimester, consistent with prior reports (21, 51, 56). This reduction may be partly explained by maternal circulating hCG levels, which have been implicated in regulating both the number and function of Bregs (72). However, no definitive set of surface markers currently exists to identify Bregs, and we align with recommendations suggesting that accurate enumeration of Bregs should rely on functional characterization, specifically, single-cell cytokine production measurements, through intracellular staining for IL-10 and IL-35 (73, 74). This functional characterization is often overlooked in many studies. We aim to complement and validate the present findings through additional functional studies, including the evaluation of cytokine secretion by both B and T cells.

T cell responses are generally believed to be suppressed during pregnancy, although the exact mechanism remains unclear. We observed several dynamic changes across various T cell subsets, including Tfh cells, which are known to mediate adaptive immune responses in various human autoimmune diseases such as rheumatoid arthritis, systemic lupus erythematosus, and multiple sclerosis (28). In contrast, the role of Tfh cells during pregnancy remains poorly defined, with most existing studies being conducted in mice. Limited research suggests that Tfh cells may support successful allogeneic pregnancy in mice (75), while in humans, a study by Monteiro and collaborators reported that human pregnancy favors Tfh cell expansion, potentially driven by elevated estrogen levels, which enhance Tfh cell differentiation and promote humoral immunity while balancing Th1/Th2 responses (76).

In our study, although no significant changes in cTfh cell levels were observed along pregnancy, the pregnant group consistently showed lower cTfh cell counts in all trimesters compared to non-pregnant women. Additionally, a significant decrease in cTfh cell percentages was noted in the third trimester. We also provide further insight into Tfh cell dynamics during pregnancy, demonstrating that both the frequency and absolute counts of activated cTfh cells expressing PD-1, CD38, and HLA-DR were reduced in pregnant women compared to non-pregnant controls. Similar findings were observed by Fröhlich et al., who characterized circulating mice CXCR5^+^PD-1^+^ICOS^+^ Tfh cells and found a decrease during the first trimester (77). They also reported lower levels of CXCR5^+^Bcl-6^+^ Tfh cells in normal pregnant mice compared to both non-pregnant and mice with disturbed pregnancies (77). Interestingly, a study by Zeng et al. using an allogeneic pregnancy mouse model found that uterine and placental CD4 T cells exhibited a Tfh-like phenotype. Specifically, CXCR5^hi^PD-1^hi^ and CXCR5hiICOS^hi^ Tfh cells with a memory/activation phenotype peaked at mid-pregnancy and were abundantly located in the uterus, with a marked increase in the placenta by late pregnancy (75). This tissue-specific redistribution of Tfh-like cells may help explain the lower circulating Tfh cell levels observed in our study. In addition, the hormonal effect in these populations still needs more consistent functional approaches to fully detail and ascertain the real impact and how the follicular functions can be modulated. In future studies, we aim to evaluate cytokine secretion by Tfh cells after stimulation to complete the current analysis.

Follicular CD8 T cells (cTfc) are a more controversial subset, poorly described, particularly in peripheric compartments, but they might play a critical role in cytokine signaling and cell-cell interactions, modulating both Tfh and B cells in humoral responses (78). To the best of our knowledge, there are no reports on this population during pregnancy. Thus, for the first time, we report the progressive decline in cTfc levels in pregnancy, with consistently lower levels across all trimesters compared to non-pregnant women. These findings suggest that both Tfh and, particularly, Tfc populations are dynamically altered during pregnancy, providing new insights into immune adaptations beyond the traditionally studied Th cell responses.

In contrast, we identified increasing proportions of non-follicular CD4 and CD8 T cell subsets expressing both activation and maturation markers throughout pregnancy, particularly PD-1^+^CD38^hi^HLA-DR^+^ and PD-1-CD38^hi^HLA-DR^+^ non-Tfc subsets. In accordance with these activated phenotypes, we observed a pregnancy-specific increase in the basal proportion of CD69^+^PD-L1^−^ and CD69^+^PD-L1^+^activated peripheral CD4 and CD8 T cells. A study in C57BL/6 mice reported elevated baseline levels of CD69^+^ CD4 T cells (but not CD8) in late pregnancy compared to non-pregnant mice (79). Similarly, Demery-Poulos et al. observed increased proportions of activated CD69^+^ peripheral CD4 T cells in third-trimester pregnant women (57). Given the presence of fetal antigens in maternal circulation, it is plausible that the increased activation of T cells in pregnancy results from repeated antigenic exposure (80, 81). Moreover, cell-free fetal DNA levels in maternal circulation rise in late pregnancy, coinciding with a pro-inflammatory shift before labor (82). Phenotyping and omics studies have provided further evidence of T cell activation during labor (83, 84), with increased activation marker expression linked to T cell responses in late pregnancy (85).

Taken together, these findings suggest that pregnancy is characterized by a gradual increase in basal activation of peripheral CD4^+^ and CD8^+^ T cells, which intensifies as labor approaches. Although no studies have directly examined these subsets in pregnancy-related pathologies, an increased CD69 expression on peripheral T cells has been reported in patients with a history of recurrent spontaneous miscarriage (86, 87). Both basal and stimulated CD69 expression were higher in women who experienced miscarriage compared to those with normal pregnancies (87). Additionally, *in vivo* activation of T cells with an anti-CD3ϵ antibody during mid-to-late pregnancy has been shown to trigger systemic inflammation, preterm labor, and birth (88), suggesting that excessive maternal T cell activation can be detrimental to fetal health. The increased proportions of activated CD8 T cells observed in our clinical dropouts may reflect these consequences. Therefore, this study establishes an important reference for future research into immune patterns during healthy pregnancy and underscores the need for further investigation in pathological cohorts.

Indeed, regular assessment of hematological and immunological profiles is a vital and standard medical practice for evaluating health status. Normal reference intervals (RIs) for these parameters in healthy populations serve as critical cutoff points for clinical decision-making. Deviations from these reference ranges can indicate disease and are essential for diagnosis and patient monitoring, particularly in the era of evidence-based medicine. Establishing baseline immunological reference ranges in healthy pregnant women is crucial for predicting and improving pregnancy outcomes during antenatal care.

In this study, we present preliminary RIs for key lymphocyte subsets, particularly those that exhibited significant changes throughout gestation compared to the non-pregnant group. To our knowledge, this is the first report to provide reference ranges for these specific immune populations throughout the entire course of pregnancy. While previous studies have established reference values for major leukocyte populations, including lymphocytes and some subsets (89, 90), more specific immune populations have remained largely uncharacterized, leaving a critical gap in the field. Given the growing body of evidence linking immune dysregulation during pregnancy with adverse outcomes (91), monitoring immune subsets whose deviations may signal impending complications is of clear clinical relevance. Although this study was not initially designed to assess the applicability of these RIs to pregnancy complications, we did observe immune deviations in participants who discontinued the study due to pregnancy-related complications. Notably, each of these individuals exhibited at least one deviation from the established RIs, suggesting a potential foundation for future evaluation of these populations in the context of pregnancy complications. Interestingly, deviations in the second-trimester evaluations were characterized by elevated levels, whereas those in the first-trimester evaluation showed reduced levels. While these findings are noteworthy, they are not statistically significant and should not be generalized to the broader population. Further validation of these reference intervals in future larger cohort studies is necessary to confirm their clinical relevance.

Additionally, we found evidence that certain immune populations altered during gestation also varied significantly with maternal age, parity, history of miscarriage, and BMI. In contrast, a previous study with a smaller sample size found no association between maternal characteristics and immune dynamics (21). Due to the limited number of participants in our study, we are unable to establish robust reference intervals stratified by demographic and clinical variables. Nonetheless, our findings highlight the importance of larger cohort studies to refine these reference intervals and to assess their clinical relevance in pregnant populations.

We acknowledge some limitations in our study. First, while our total sample size (n=50) is comparable to that of other studies in literature, it limited our ability to conduct detailed subgroup analyses, thereby reducing the statistical robustness of some stratified comparisons. Additionally, incomplete data collection during the second and third trimesters may have impacted the completeness of the longitudinal dataset. While our cohort included only women who delivered at term, participants varied in demographic and clinical characteristics, which may have influenced immune characteristics and contributed to interindividual variability. Nonetheless, we successfully captured chronological immune adaptations throughout term pregnancies. Another limitation is the recruitment of participants from a single private healthcare center, which may restrict the generalizability of our findings. Future research should investigate how ethnic, racial, and socioeconomic factors influence immune adaptations during pregnancy. Regarding the establishment of reference interval, although we did not meet the recommended minimum sample size (n=120), we employed the robust statistical method recommended by the CLSI for smaller datasets. While validated, this approach would benefit from replication in larger cohorts to enhance the clinical utility of the proposed reference intervals. Finally, future studies incorporating more representative cohorts and postpartum follow-up will be essential to confirm the reproducibility of our findings and to further elucidate immune adaptations during and after pregnancy.

Despite these limitations, our study presents several strengths. Unlike many similar studies, we conducted a longitudinal follow-up of the same participants across defined gestational timepoints, thereby reducing cohort heterogeneity and enhancing internal validity. In addition to analyzing immune cell population frequencies and absolute cell counts, we also assessed cellular functional states and responsiveness, an aspect often overlooked in comparable research. Furthermore, our study is among the few to perform a longitudinal analysis of immune adaptations during pregnancy using a well-defined and pre-established timeline and a standardized, optimized methodology. Another key strength is our inclusion of effect size calculation, which enhances both the interpretability and reliability of our findings. Unlike p-values, which only indicate statistical significance, effect sizes quantify the magnitude of differences or associations, offering deeper insight into their biological and clinical relevance (35, 92). This is particularly important in studies with smaller sample sizes, where statistically significant results may not always reflect meaningful effects. Additionally, effect size calculations facilitate comparisons with previous research, allowing for better contextualization within the broader scientific literature. By incorporating these analyses, we strengthen the interpretative value of our results and increase the study’s potential impact in the field.

## Conclusion

This study provides a detailed characterization of immune changes throughout gestation, offering novel insights into the immune subsets influenced by pregnancy and their functional capacities. These findings contribute to a more comprehensive understanding of systemic maternal immune adaptations during pregnancy. In addition, we present pregnancy-specific reference intervals for key immune cell subsets, which may serve as a valuable resource for both future research and potential clinical applications. Notably, the immune deviations observed in participants who experienced pregnancy complications underscore the importance of establishing additional reference values and highlight the need for validation in larger, independent cohorts.

## Data Availability

The original contributions presented in the study are included in the article/**Supplementary Material**. Further inquiries can be directed to the corresponding author.

## Supplementary material

Supplementary Figure 1 Representative gating for B cell populations identified by the B cell panel of the flow cytometry analysis. Initial gating was performed with Infinicyt software to isolate the lymphocyte population (CD45^+^SSC^low^FSC^low^) and exclusion of doublets and artifacts (Black box). Files were then exported, and subsequent analysis was performed using FlowJo software (Red box)

Supplementary Figure 2 Representative gating for CD4 T cell populations identified by the T cell panel of the flow cytometry analysis. Initial gating was performed with Infinicyt software to isolate the lymphocyte population (CD45^+^SSC^low^FSC^low^) and exclusion of doublets and artifacts (Black box). Files were then exported, and subsequent analysis was performed using FlowJo software (Red box).

Supplementary Figure 3 Representative gating for CD8 T cell populations identified by the T cell panel of the flow cytometry analysis. Initial gating was performed with Infinicyt software to isolate the lymphocyte population (CD45^+^SSC^low^FSC^low^) and exclusion of doublets and artifacts (black box). Files were then exported, and subsequent analysis was performed using FlowJo software (red box).

Supplementary Figure 4 Representative gating for CD69- and PD-L1-expressing B, CD8^-^ and CD8^+^ T cell populations. Analysis was performed using FlowJo software

Supplementary Figure 5 Longitudinal changes in erythrogram components during pregnancy. Values of main erythrogram components across pregnancy and non-pregnant group. The mean with standard deviation of the indicated parameter across groups is indicated, and the normal range is represented with shaded areas. Statistical significance between trimesters was determined with mixed-effects analysis followed by Tukey’s multiple comparisons test and are indicated as: **p* < 0.05, **p < 0.01, ***p < 0.001, *****p* < 0.0001. Comparisons between pregnant and non-pregnant groups were performed with ordinary One-way ANOVA followed by Dunnett’s multiple comparison test and are indicated as †*p* < 0.05, ††p < 0.01, †††p< 0.001, ††††*p* < 0.0001. RBC, Red blood cells; Hg, Hemoglobin; Hct, Hematocrit; MCV, Mean Corpuscular Volume; MCH, Mean Corpuscular Hemoglobin; MCHC, Mean Corpuscular Hemoglobin Concentration; RDW, Red Cell Distribution Width; Hypo, Hypochromic.

Supplementary Figure 6 Changes in absolute counts of peripheral blood lymphocytic populations in pregnant and non-pregnant groups. **(A)** Longitudinal comparisons between the 1^st^ (T1), 2^nd^ (T2), and 3^rd^ (T3) trimester study visits. **(B)** Comparisons between non-pregnant women (NPW) and each trimester of the pregnant group. Only subsets of immune cell populations with a fold change greater than 1.1 or less than 0.9 and significant differences for at least one comparison are represented. Only subsets with more than 5 cells/ul in at least one group were considered for analysis. Statistical significance was calculated using Tukey’s **(A)** or Dunnett’s **(B)** multiple comparisons test and are denoted as follows: *p < 0.05, **p < 0.01, ***p < 0.001, and ****p < 0.0001. The colour gradient represents fold change on a Log_2_ scale of each subset’s absolute count (0 = no change). Detailed information is available in **Supplementary Table 4**.
