# Supplementary figures and images for: Immunological reference intervals in pregnancy: longitudinal analysis of adaptive lymphocyte subsets
Source: Front Immunol. 2025 Sep 17;16:1634176. doi: 10.3389/fimmu.2025.1634176 (PMC12483863; doi:10.3389/fimmu.2025.1634176)

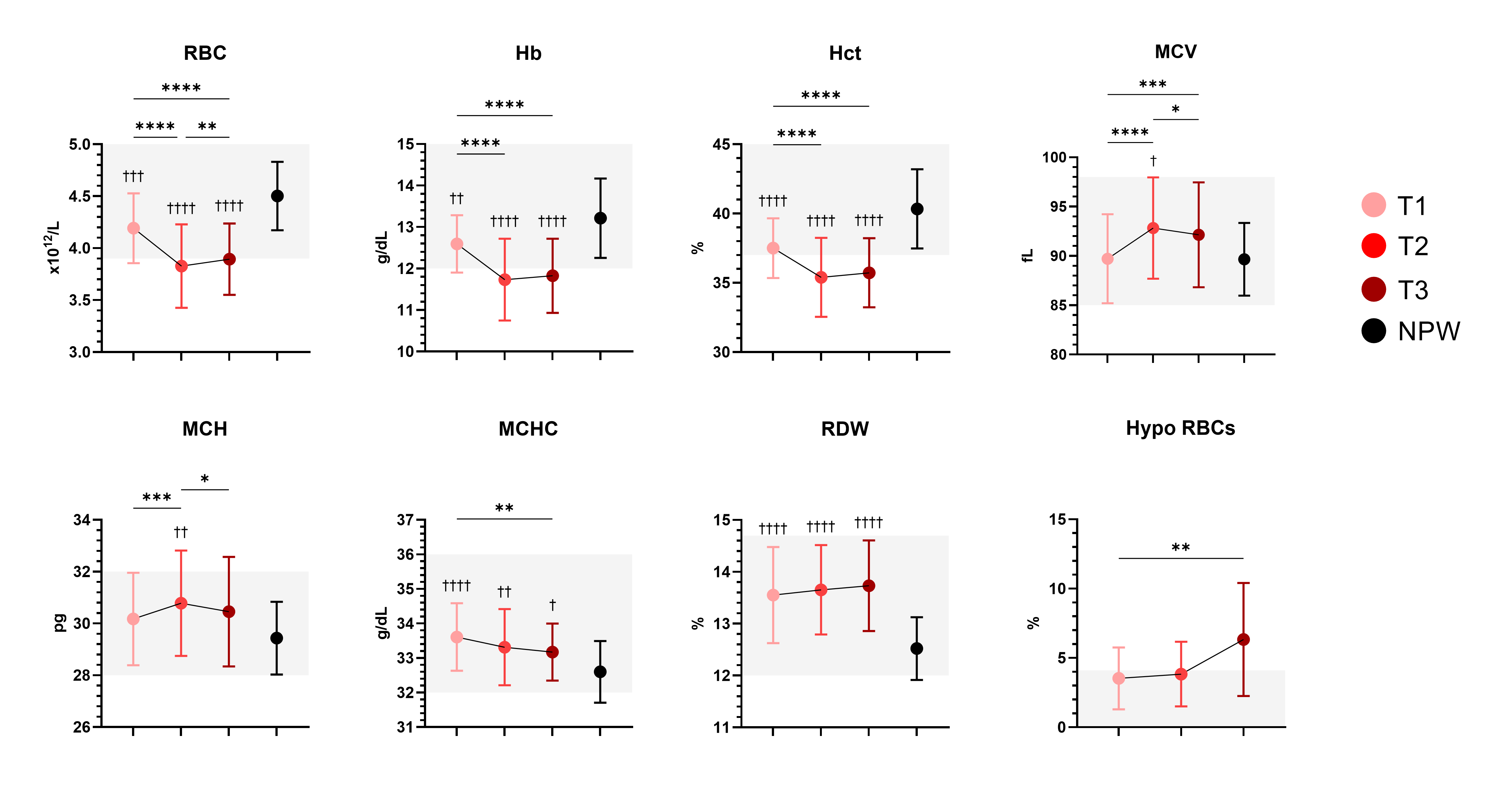

Supplement: Supplementary Figure 1 — Representative gating for B cell populations identified by the B cell panel of the flow cytometry analysis. Initial gating was performed with Infinicyt software to isolate the lymphocyte population (CD45+SSClowFSClow) and exclusion of doublets and artifacts (Black box). Files were then exported, and subsequent analysis was performed using FlowJo software (Red box) [file Image1.tif]

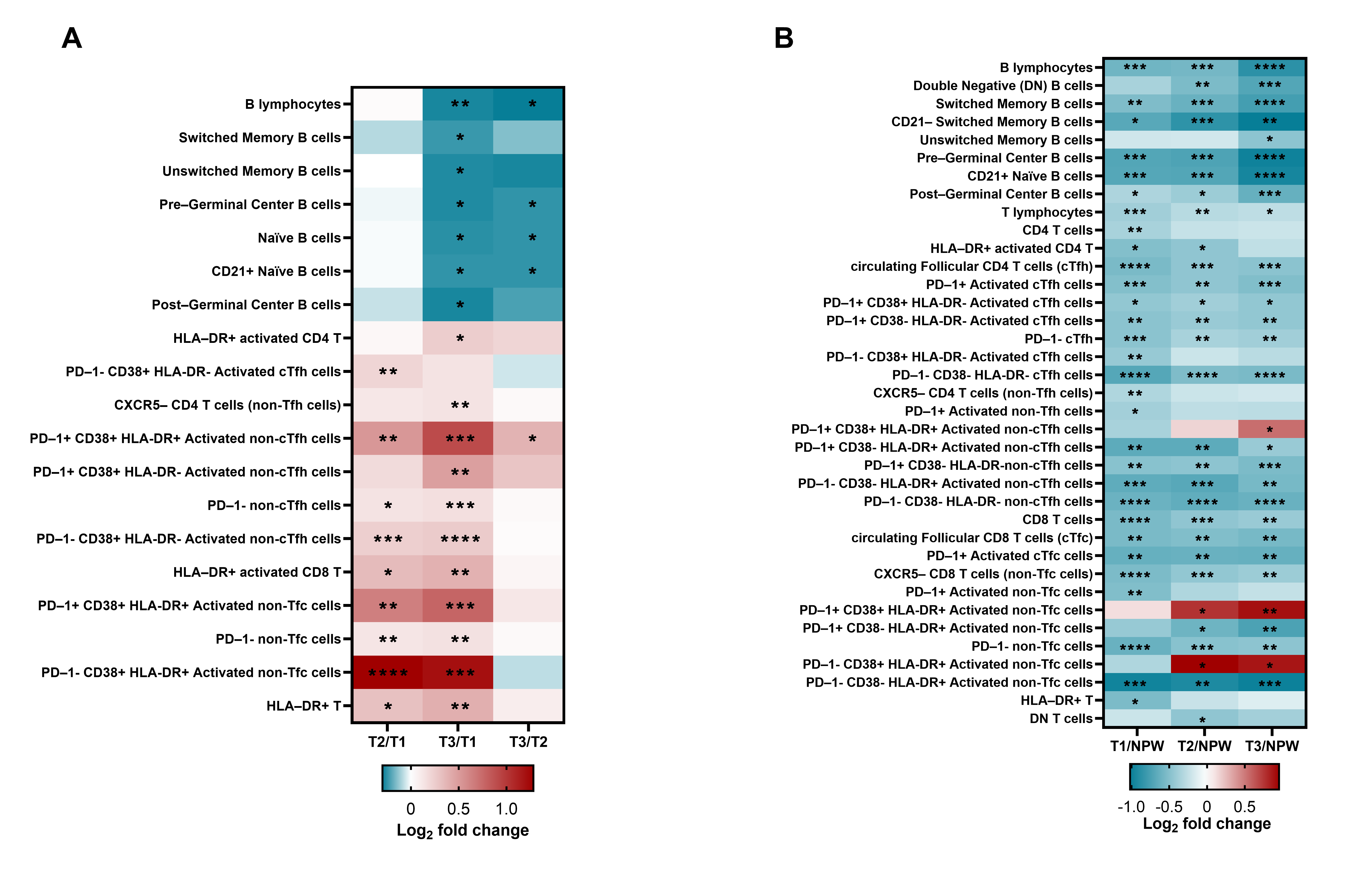

Supplement: Supplementary Figure 2 — Representative gating for CD4 T cell populations identified by the T cell panel of the flow cytometry analysis. Initial gating was performed with Infinicyt software to isolate the lymphocyte population (CD45+SSClowFSClow) and exclusion of doublets and artifacts (Black box). Files were then exported, and subsequent analysis was performed using FlowJo software (Red box). [file Image2.tif]

# B cell gating

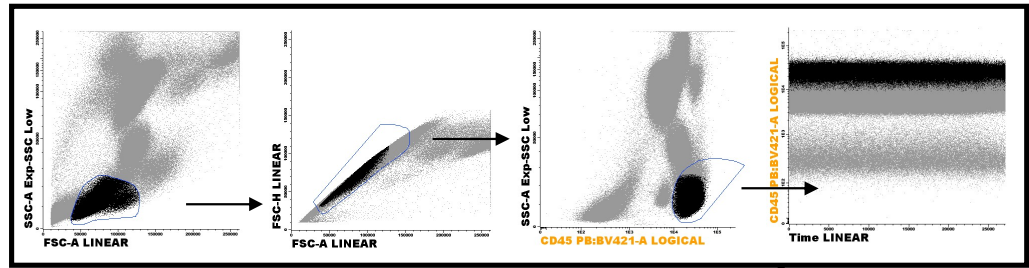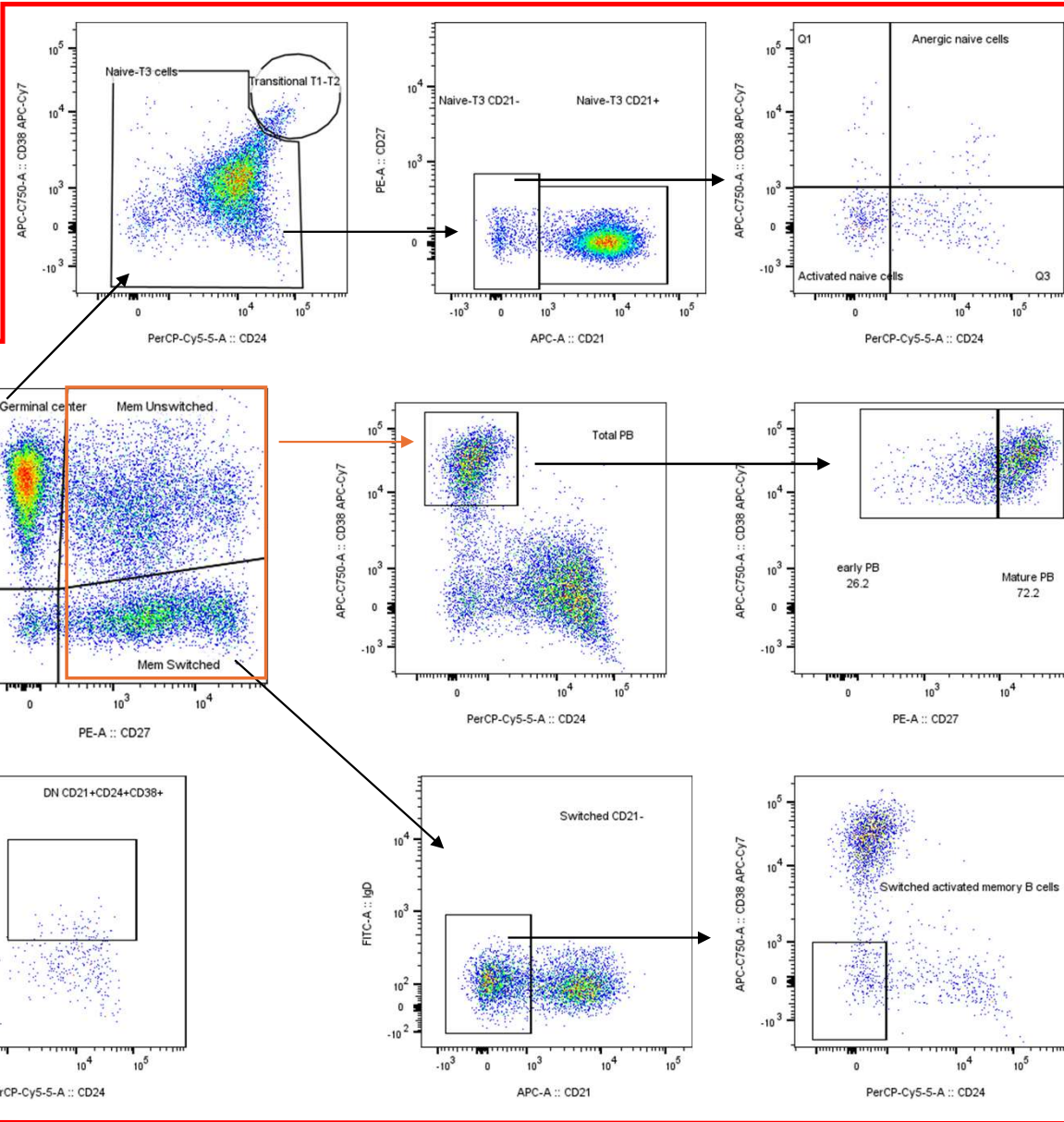

Supplement: Supplementary Figure 3 — Representative gating for CD8 T cell populations identified by the T cell panel of the flow cytometry analysis. Initial gating was performed with Infinicyt software to isolate the lymphocyte population (CD45+SSClowFSClow) and exclusion of doublets and artifacts (black box). Files were then exported, and subsequent analysis was performed using FlowJo software (red box). [file DataSheet1.pdf]

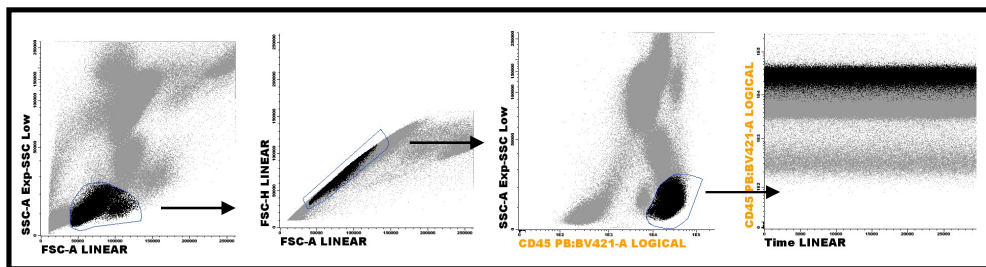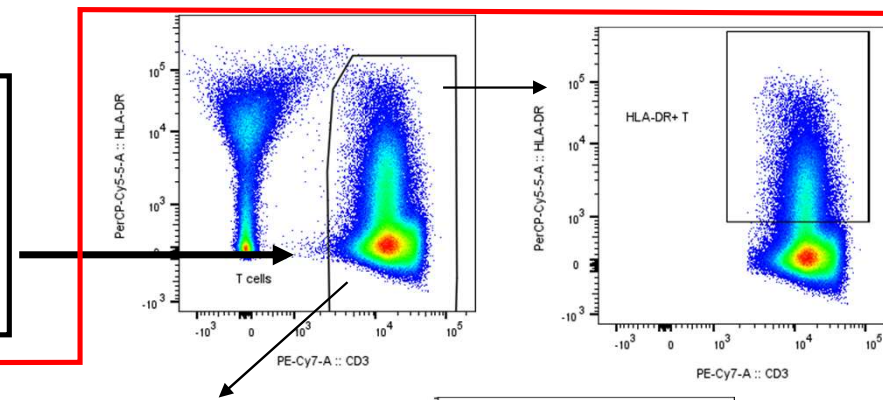

CD4 T cell gating

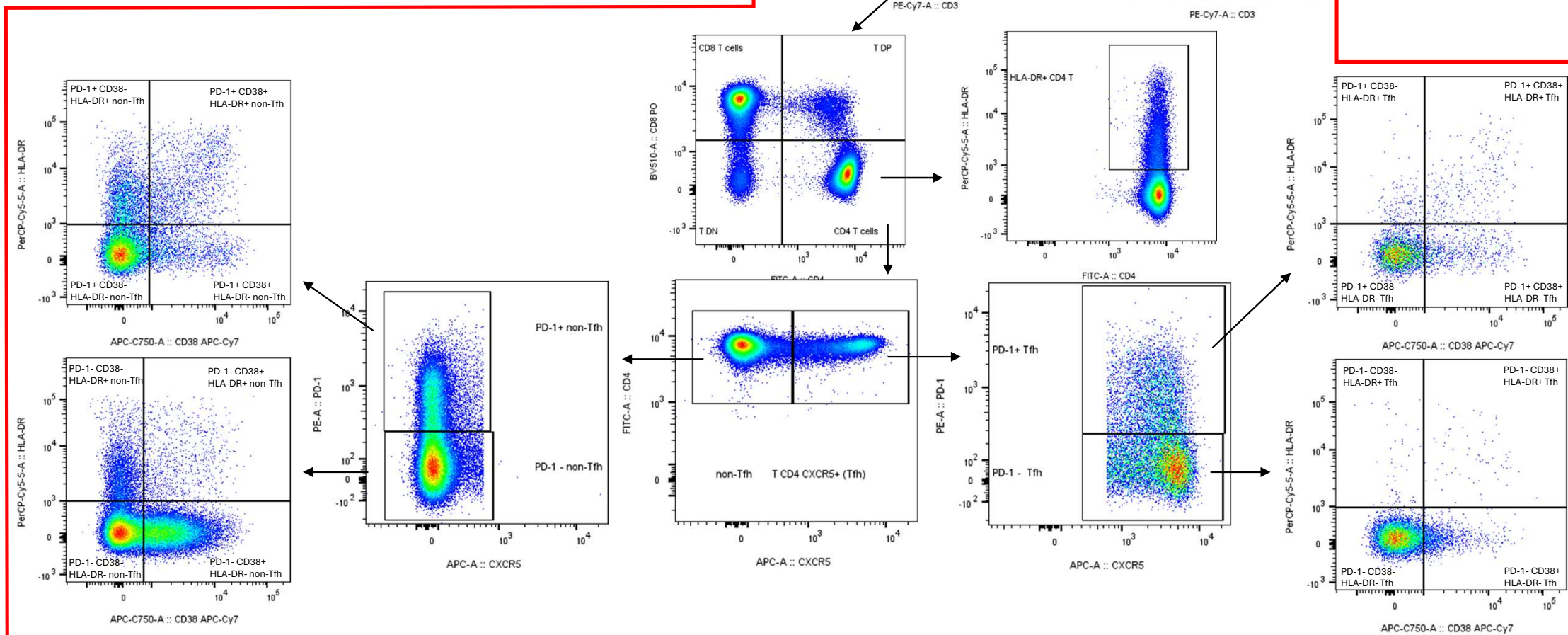

Supplement: Supplementary Figure 4 — Representative gating for CD69- and PD-L1-expressing B, CD8- and CD8+ T cell populations. Analysis was performed using FlowJo software [file DataSheet2.pdf]

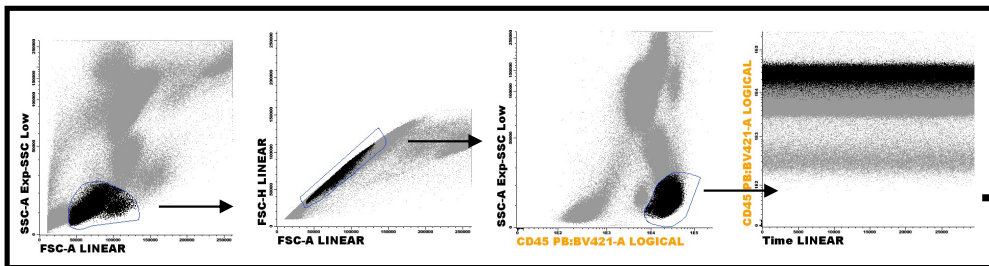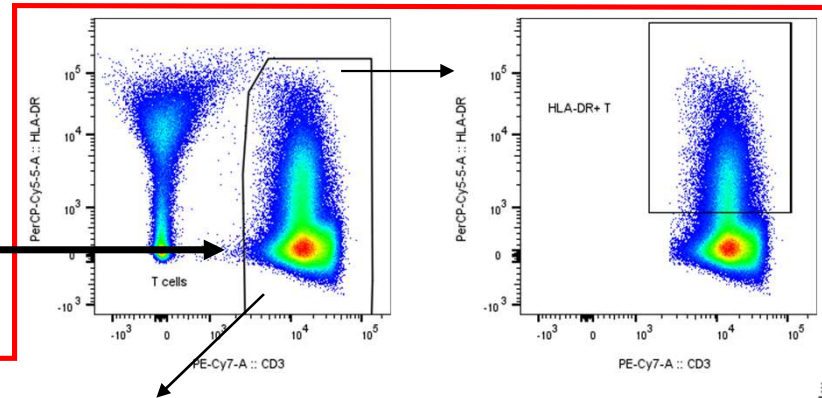

CD8 T cell gating

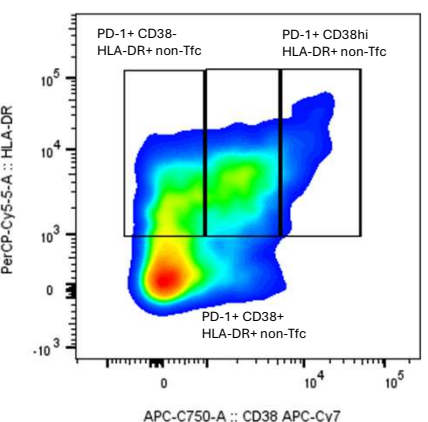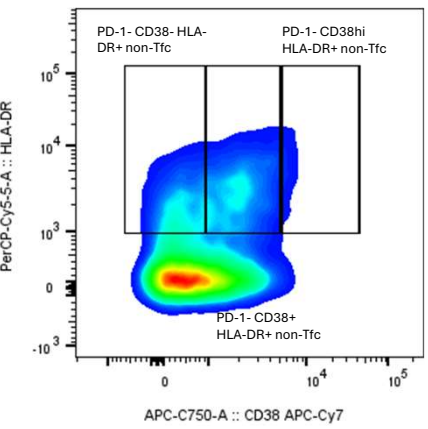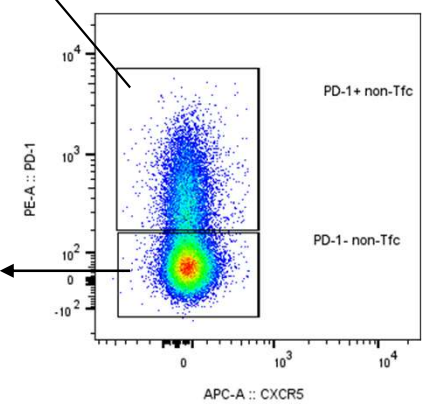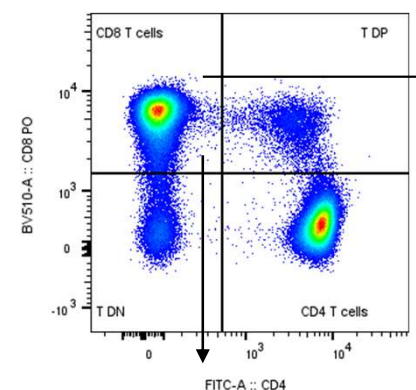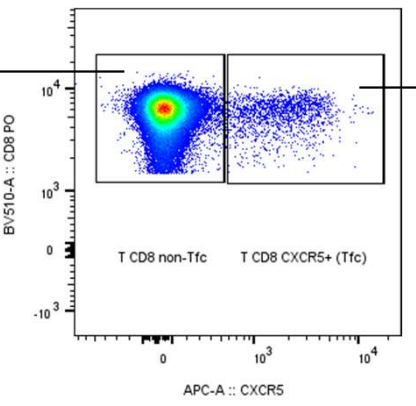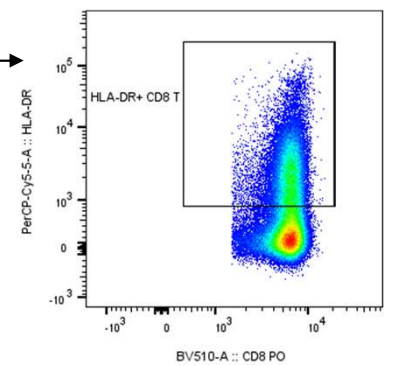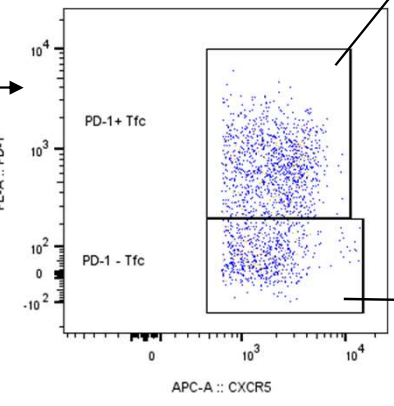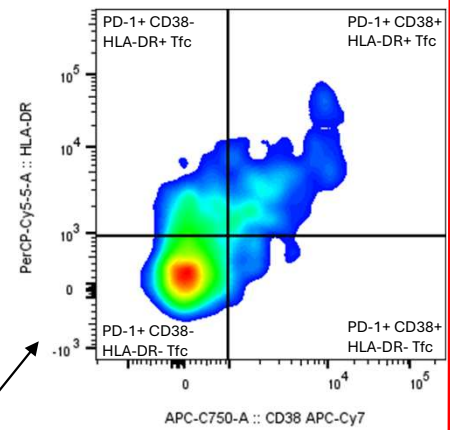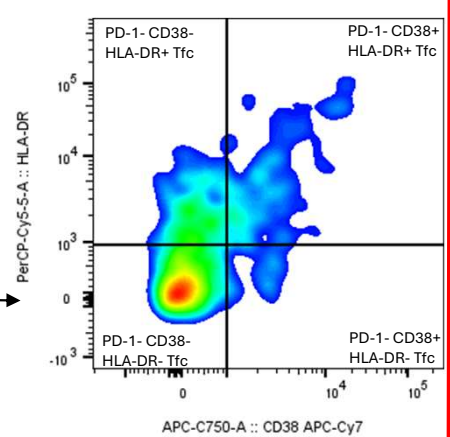

Supplement: Supplementary Figure 5 — Longitudinal changes in erythrogram components during pregnancy. Values of main erythrogram components across pregnancy and non-pregnant group. The mean with standard deviation of the indicated parameter across groups is indicated, and the normal range is represented with shaded areas. Statistical significance between trimesters was determined with mixed-effects analysis followed by Tukey’s multiple comparisons test and are indicated as: *p < 0.05, **p < 0.01, ***p < 0.001, ****p < 0.0001. Comparisons between pregnant and non-pregnant groups were performed with ordinary One-way ANOVA followed by Dunnett’s multiple comparison test and are indicated as †p < 0.05, ††p < 0.01, †††p< 0.001, ††††p < 0.0001. RBC, Red blood cells; Hg, Hemoglobin; Hct, Hematocrit; MCV, Mean Corpuscular Volume; MCH, Mean Corpuscular Hemoglobin; MCHC, Mean Corpuscular Hemoglobin Concentration; RDW, Red Cell Distribution Width; Hypo, Hypochromic. [file DataSheet3.pdf]

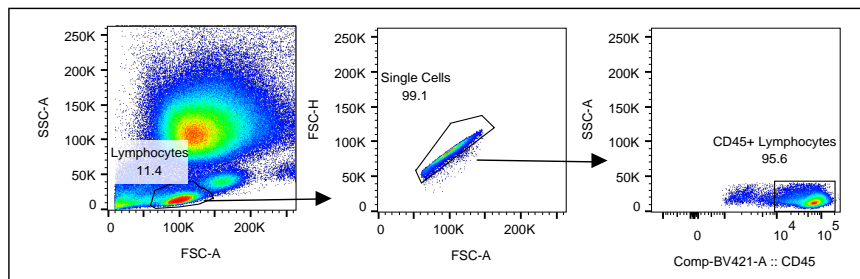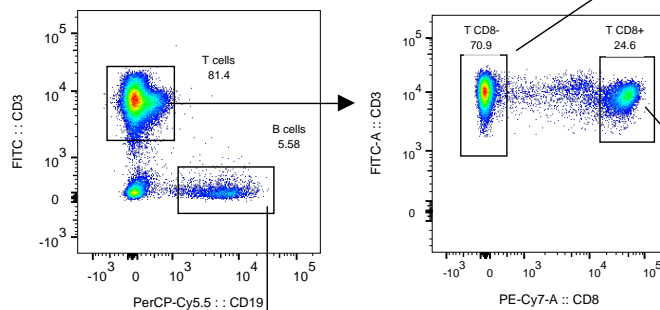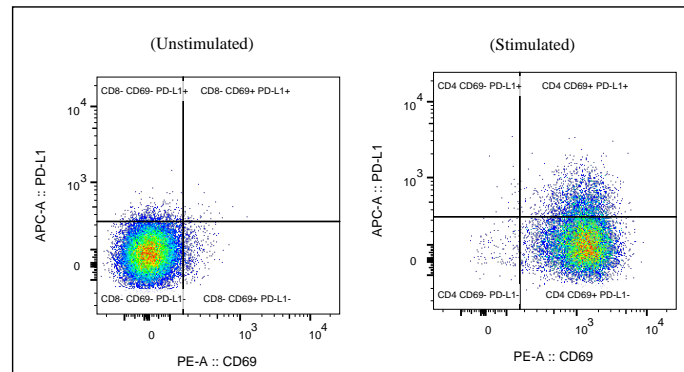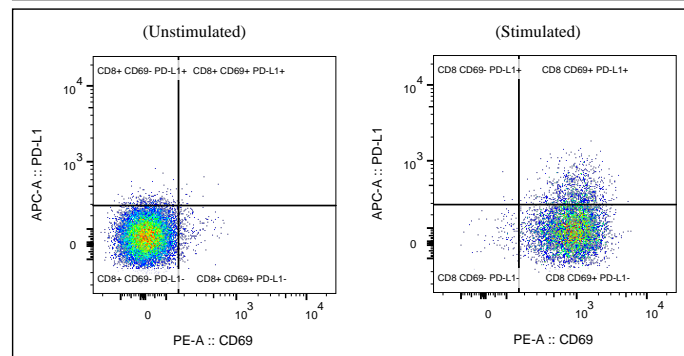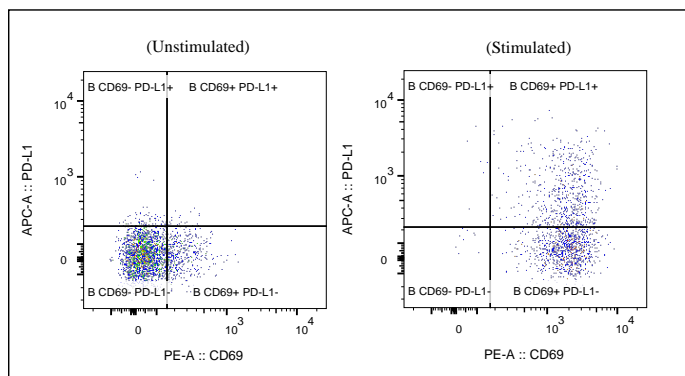

Supplement: Supplementary Figure 6 — Changes in absolute counts of peripheral blood lymphocytic populations in pregnant and non-pregnant groups. (A) Longitudinal comparisons between the 1st (T1), 2nd (T2), and 3rd (T3) trimester study visits. (B) Comparisons between non-pregnant women (NPW) and each trimester of the pregnant group. Only subsets of immune cell populations with a fold change greater than 1.1 or less than 0.9 and significant differences for at least one comparison are represented. Only subsets with more than 5 cells/ul in at least one group were considered for analysis. Statistical significance was calculated using Tukey’s (A) or Dunnett’s (B) multiple comparisons test and are denoted as follows: *p < 0.05, **p < 0.01, ***p < 0.001, and ****p < 0.0001. The colour gradient represents fold change on a Log2 scale of each subset’s absolute count (0 = no change). Detailed information is available in Supplementary Table 4 . [file DataSheet4.pdf]
